# Supplementary material for: Combinatorial protein engineering identifies potent CRISPR activators with reduced toxicity
Source: Nat Commun. 2025 Nov 20;16:11114. doi: 10.1038/s41467-025-65986-4 (PMC12706070; doi:10.1038/s41467-025-65986-4)
Supplement: Supplementary file 1 — Reporting Summary [file 41467_2025_65986_MOESM1_ESM.pdf]

nature portfolio

Corresponding author(s): Alejandro Chavez and Lai Wei

Last updated by author(s): Oct 17, 2025

Reporting Summary

Nature Portfolio wishes to improve the reproducibility of the work that we publish. This form provides structure for consistency and transparency in reporting. For further information on Nature Portfolio policies, see our Editorial Policies and the Editorial Policy Checklist.

Statistics

For all statistical analyses, confirm that the following items are present in the figure legend, table legend, main text, or Methods section.

n/a	Confirmed	
☐	☒	The exact sample size (*n*) for each experimental group/condition, given as a discrete number and unit of measurement
☐	☒	A statement on whether measurements were taken from distinct samples or whether the same sample was measured repeatedly
☐	☒	The statistical test(s) used AND whether they are one- or two-sided<br>*Only common tests should be described solely by name; describe more complex techniques in the Methods section.*
☐	☒	A description of all covariates tested
☒	☐	A description of any assumptions or corrections, such as tests of normality and adjustment for multiple comparisons
☐	☒	A full description of the statistical parameters including central tendency (e.g. means) or other basic estimates (e.g. regression coefficient) AND variation (e.g. standard deviation) or associated estimates of uncertainty (e.g. confidence intervals)
☐	☒	For null hypothesis testing, the test statistic (e.g. *F*, *t*, *r*) with confidence intervals, effect sizes, degrees of freedom and *P* value noted<br>*Give P values as exact values whenever suitable.*
☒	☐	For Bayesian analysis, information on the choice of priors and Markov chain Monte Carlo settings
☒	☐	For hierarchical and complex designs, identification of the appropriate level for tests and full reporting of outcomes
☐	☒	Estimates of effect sizes (e.g. Cohen's *d*, Pearson's *r*), indicating how they were calculated

Our web collection on statistics for biologists contains articles on many of the points above.

Software and code

Policy information about availability of computer code

Data collection	MaxQuant (version 4.8.7), MSstats (version 4.14.0), Bioconductor (version 1.18.0), using SAINTexpress (version 3.6.3), psych (version 2.4.3), bcl2fastq2 (version 2.20), Cutadapt (version 2.1), Kallisto (version 0.44.0), BioJupies (RRID:SCR_016346), PADDLE
Data analysis	The code supporting the findings of this study is available under the MIT license, on Zenodo at DOI 10.5281/zenodo.10962794 [https://doi.org/10.5281/zenodo.10962793].

For manuscripts utilizing custom algorithms or software that are central to the research but not yet described in published literature, software must be made available to editors and reviewers. We strongly encourage code deposition in a community repository (e.g. GitHub). See the Nature Portfolio guidelines for submitting code & software for further information.

Data

Policy information about availability of data

All manuscripts must include a data availability statement. This statement should provide the following information, where applicable:

- Accession codes, unique identifiers, or web links for publicly available datasets
- A description of any restrictions on data availability
- For clinical datasets or third party data, please ensure that the statement adheres to our policy

The next-generation sequencing datasets generated and analyzed in this study are available in the NCBI Sequence Read Archive (SRA) under BioProject accession number PRJNA1065930 [https://www.ncbi.nlm.nih.gov/bioproject/PRJNA1065930]. Plasmids expressing MHV, MMH, MPH, dCas9 and dCas9-VP64 have been deposited to Addgene [https://www.addgene.org/browse/article/28247691/]. The mass spectrometry proteomics data have been deposited to the

ProteomeXchange Consortium via the PRIDE partner repository with the dataset identifier PXD068296 [https://proteomecentral.proteomexchange.org/cgi/GetDataset?ID=PXD068296]. Source data are provided with this paper (Source Data.zip).

Research involving human participants, their data, or biological material

Policy information about studies with human participants or human data. See also policy information about sex, gender (identity/presentation), and sexual orientation and race, ethnicity and racism.

Reporting on sex and gender	No human participants were involved in this study.
Reporting on race, ethnicity, or other socially relevant groupings	See above.
Population characteristics	See above.
Recruitment	See above.
Ethics oversight	See above.

Note that full information on the approval of the study protocol must also be provided in the manuscript.

Field-specific reporting

Please select the one below that is the best fit for your research. If you are not sure, read the appropriate sections before making your selection.

[x] Life sciences [ ] Behavioural & social sciences [ ] Ecological, evolutionary & environmental sciences

For a reference copy of the document with all sections, see nature.com/documents/nr-reporting-summary-flat.pdf

Life sciences study design

All studies must disclose on these points even when the disclosure is negative.

Sample size	Initial experiments tested 230 individual ADs to identify strong performers. From these, 25 domains were selected for combinatorial assembly into bipartite and tripartite constructs – balancing the goal of exploring a broad combinatorial space (25^3 = 15,625 tripartite constructs) with the practical requirements of maintaining sufficient library representation in cell culture. Three targets (EPCAM, CXCR4, and a synthetic reporter) were chosen to gauge target specificity while keeping the screening effort tractable. No statistical method was used to predetermine sample size. Screening experiments were performed in duplicate (n = 2 independent biological replicates), a sample size chosen to balance reproducibility with the practical demands of maintaining representation across thousands of constructs per screen. Manual validation experiments were also performed in duplicate (n = 2 independent biological replicates), consistent with prior CRISPR activator studies reporting head-to-head comparisons. Mass spectrometry experiments were performed in quadruplicate (n = 4 independent biological replicates) to ensure robust protein interaction profiling. In all cases, replicate experiments produced concordant results, supporting the reliability of our findings.
Data exclusions	Certain tripartite activators were excluded from analyses because they failed to meet designated read minimums. These activators were not well enough represented in cells to enable confident analysis of their behavior. No single-domain or bipartite activators were excluded from analyses. We believe tripartite activators displayed greater droupout in cells due to higher toxicity levels.<br><br>We also excluded domain A24, mannose binding protein, originally included in the library as an inert protein folder, from all analyses utilizing protein folders because it was found to negatively impact activator behavior.
Replication	All experiments (one-by-one activation experiments and pooled activation screens) were performed in biological duplicate, except our mass spec analyses, which were performed in biological quadruplicate. For all experiments, we calculated Pearson correlations across biological replicates to ensure that our data was high quality. All attempts at replication were successful.
Randomization	This is not relevant to our study. Given the nature of the pooled screening approach, all combinations were evaluated in the same way, ensuring uniformity in the testing conditions. Covariate control was inherently managed by the uniformity of the experimental setup. Since all AD combinations were exposed to the same cellular conditions, same vector systems, and same target gene context, the effect of external covariates was minimized. This uniform approach ensures that any observed differences in gene activation are attributable solely to the inherent efficacy of each AD combination rather than external experimental variables.
Blinding	Blinding was not relevant to our study because the outcomes were based on assays whose outcomes did not depend on subjective interpretation. The experimental setup involved direct, quantifiable data from biological samples, minimizing the potential for bias and negating the need for blinding.

Reporting for specific materials, systems and methods

We require information from authors about some types of materials, experimental systems and methods used in many studies. Here, indicate whether each material, system or method listed is relevant to your study. If you are not sure if a list item applies to your research, read the appropriate section before selecting a response.

Materials & experimental systems

n/a	Involved in the study
☐	☒ Antibodies
☐	☒ Eukaryotic cell lines
☒	☐ Palaeontology and archaeology
☒	☐ Animals and other organisms
☒	☐ Clinical data
☒	☐ Dual use research of concern
☒	☐ Plants

Methods

n/a	Involved in the study
☒	☐ ChIP-seq
☐	☒ Flow cytometry
☒	☐ MRI-based neuroimaging

Antibodies

Antibodies used

The following antibodies were used. Each was diluted 1:100, and 100 µL of the diluted antibody solution was used to resuspend each cell pellet: anti-human EPCAM-PE (clone VU-1D9, Invitrogen, Cat. #A15782), anti-human CXCR4-PE (clone 12G5, BioLegend, Cat. #306506), anti-human CD2-APC/Cyanine7 (clone RPA-2.10, BioLegend, Cat. #300219), anti-human CD45-PerCP-Cy5.5 (clone QA17A19, BioLegend, Cat. #393419), anti-human EPCAM-FITC (clone VU-1D9, Invitrogen, Cat. #A15755), anti-human EGFR-Alexa Fluor 647 (clone AY13, BioLegend, Cat. #352918), and anti-human CXCR4-Alexa Fluor 594 (clone 44716, R&D Biotechne, Cat. #FAB172T-100UG).

Validation

Antibody validation was performed as described in the Antibody Staining and Flow Cytometry section of the Methods. Briefly, each antibody was tested by staining cells in which the target protein was synthetically activated and comparing signal to non-activated controls, confirming specificity and functionality. All antibodies were used according to the manufacturers' instructions.

Eukaryotic cell lines

Policy information about cell lines and Sex and Gender in Research

Cell line source(s)

HEK293T cells, Hela cells (both gifts of P. Mali, UCSD, San Diego, CA), HCT116 cells (ATCC CCL-247), and N2A cells (ATCC CCL-131) were used in this study.

Authentication

None of the cell lines used were directly authenticated by our group.

Mycoplasma contamination

All cell lines tested negative for mycoplasma contamination.

Commonly misidentified lines
(See ICLAC register)

No commonly misidentified cell lines were used in this study.

Plants

Seed stocks

Not applicable.

Novel plant genotypes

Not applicable.

Authentication

Not applicable.

Flow Cytometry

Plots

Confirm that:

☒ The axis labels state the marker and fluorochrome used (e.g. CD4-FITC).

☒ The axis scales are clearly visible. Include numbers along axes only for bottom left plot of group (a 'group' is an analysis of identical markers).

☒ All plots are contour plots with outliers or pseudocolor plots.

☒ A numerical value for number of cells or percentage (with statistics) is provided.

Methodology

Sample preparation

Activator-expressing HEK293T or HeLa cells were harvested using 0.05% trypsin, followed by washing with PBS, staining with

	target-specific antibodies (diluted 1:100 in PBS) for one hour, and resuspending in PBS + 2% FBS.
Instrument	BD Biosciences BD LSR II, BD Biosciences BD Influx cell sorter,
Software	BD FACSDiva to collect data, FlowJo to analyze data
Cell population abundance	7,500,000, 9,000,000, or 21,000,000 cells per replicate per target gene were harvested for the single-domain, bipartite, and tripartite screens, respectively, followed by washing with PBS, staining with target-specific antibodies for one hour (for endogenous targets), and resuspending in PBS + 2% FBS. Cells were sorted into four bins of target expression. Each bin consisted of 12.5% of the population and covered only the extremes of the population (two bins covering the lower extremes, two bins covering the higher extremes) 300,000, 600,000 or 1,400,000 cells were sorted into each bin for the single-domain, bipartite, and tripartite screens, respectively. For each bin, a small portion of collected cells was re-flowed to ensure purity of the population.
Gating strategy	For individual activation experiments and pooled screens, SSC-A vs. FSC-A plots were used to gate on live cells (outliers that did not occupy the dense, middle portion of cells were excluded from the gate). For individual activation experiments SSC-H vs. SSC-A was used to gate on single cells (outliers that fell below the main linear segment of cells were excluded from the gate). For pooled screen experiments, trigger pulse width vs. FSC-A was used to gate on single cells (outliers that fell above the main linear segment of cells were excluded from the gate). For individual activation experiments, BFP was used as a transfection marker. For pooled screens targeting the synthetic reporter, iRFP was used as a transfection marker. In both instances, a negative control sample was used to determine where on the log scale x-axis (corresponding to the fluorescence intensity of the transfection marker) to draw a gate between untransfected and transfected cells.

☒ Tick this box to confirm that a figure exemplifying the gating strategy is provided in the Supplementary Information.
