# Supplementary material for: Combinatorial protein engineering identifies potent CRISPR activators with reduced toxicity
Source: Nat Commun. 2025 Nov 20;16:11114. doi: 10.1038/s41467-025-65986-4 (PMC12706070; doi:10.1038/s41467-025-65986-4)
Supplement: Supplementary file 2 — Supplementary Information [file 41467_2025_65986_MOESM2_ESM.pdf]

Combinatorial Protein Engineering Identifies Potent CRISPR Activators with Reduced Toxicity

Marla Giddins[1,2,3*], Alexander F. Kratz[2,4*], Mark B. De Los Santos[2,5,6], Antoine Forget[7,8], Richa Tiwari[7,8], Gwendolyn Jang[7,8], Tomasz Blazejewski[3,4], Chuyan Qin[9,10], Yiming Huang[3,4], Yeh-Hsing Lao[11,12], Thomas Falconer[2,13], Kam. W. Leong[3,11], Nevan Krogan[7,8,14], Max Staller[15,16,17], Harris Wang[2,3], Lai Wei[2,18†,‡] & Alejandro Chavez[2,18†,‡]

Affiliations

[1]Department of Microbiology and Immunology, Columbia University Irving Medical Center, New York, NY, 10032, USA.
[2]Department of Pathology and Cell Biology, Columbia University Irving Medical Center, New York, NY, USA.
[3]Department of Systems Biology, Columbia University Irving Medical Center, New York, NY, 10032, USA.
[4]Integrated Program in Cellular, Molecular, and Biomedical Studies, Columbia University Irving Medical Center, New York, NY, 10032, USA.
[5]Neurobiology and Behavior Program, Columbia University Irving Medical Center, New York, NY, 10032, USA.
[6]Department of Genetics and Development, Columbia University Irving Medical Center, New York, NY, 10032, USA.
[7]Quantitative Biosciences Institute (QBI), University of California, San Francisco, San Francisco, CA, USA.
[8]Department of Bioengineering and Therapeutic Sciences, University of California San Francisco, San Francisco, CA, USA.
[9]Department of Biochemistry, University of Cambridge, Tennis Court Road, Cambridge CB2 1GA, UK
[10]The Gurdon Institute, University of Cambridge, Tennis Court Road, Cambridge CB2 1QN, UK.
[11]Department of Biomedical Engineering, Columbia University, New York, New York 10027, USA.
[12]Department of Pharmaceutical Sciences, University at Buffalo, The State University of New York, Buffalo, New York 14214, USA.
[13]Department of Biomedical Informatics, Columbia University Irving Medical Center, New York, NY, 10032, USA.
[14]Gladstone Institutes, San Francisco, California, USA.
[15]Department of Molecular and Cell Biology, University of California Berkeley, Berkeley, CA 94720, USA.
[16]Center for Computational Biology, University of California Berkeley, Berkeley, CA 94720, USA.
[17]Chan Zuckerberg Biohub-San Francisco, San Francisco, CA 94158, USA.
[18]Department of Pediatrics, University of California San Diego, La Jolla, CA, 92093, USA.

*These authors contributed equally to this work.
†Correspondence: Alejandro Chavez (chavez2@health.ucsd.edu); Lai Wei (larrywei@health.ucsd.edu)
‡These authors jointly supervised this work.

Table of Contents

Supplementary Notes 1-3: Pages 2-5
Supplementary Tables 1-4: Pages 6-9
Supplementary Figures 1-36: Pages 10-47

Supplementary Notes

Supplementary Note 1. Performance of built-in screening controls

Across screens, supernatant control barcodes and multi-copy control barcodes showed significantly fewer reads than mutant MCP control barcodes (**Supplementary Figure 37**). For the single-domain screen, supernatant control barcodes and multi-copy control barcodes showed, on average, 99.9% fewer reads and 89.4% fewer reads, respectively, than mutant MCP control barcodes. For the bipartite screen, supernatant control barcodes and multi-copy control barcodes showed, on average, 80.8% fewer reads and 42.5% fewer reads, respectively, than mutant MCP control barcodes. For the tripartite screen, supernatant control barcodes and multi-copy control barcodes showed, on average, 99.8% fewer reads and 90.1% fewer reads, respectively, than mutant MCP control barcodes. Given that supernatant controls and multi-copy controls were spiked into each library at the same concentration as MCP mutant controls, these results suggest that only a small proportion of NGS reads derive from multi-copy integration events or DNA in the supernatant. These phenomena proved somewhat more prevalent within bipartite screens but did not significantly influence resultant data, based on downstream quality control (biological replate correlations and experimental validation of screen-derived activation).

Mutant MCP control constructs ranked, on average, 25.3 out of 26 in the single-domain screen, 622 out of 626 in the bipartite screen, and 817.33 out of 838 (of the set of constructs ranked across all three targets) respectively, in the tripartite screen. Our findings that this construct, which contains an inactive MCP, ranks towards the bottom of all constructs across screens highlights the accuracy of our high-throughput approach for scoring activators.

Of note, to evaluate the prevalence and scores of control constructs, each of which was appended with only a single UMI (rather than multiple UMI's, like all other activators), we carried out a separate computational pipeline that did not discard specific UMIs associated with the controls (despite their high read counts, which would have qualified them for exclusion in our non-control pipeline) and decreased the minimum read threshold required for analysis such that, upon normalization, a similar number of total activators would be quantified as in our non-control pipeline. Our computational pipeline for scoring activators without controls, used for all subsequent analyses and experimental workup, is outlined in NGS analyses, UMI filtration, and elucidation of screen activation scores Methods.

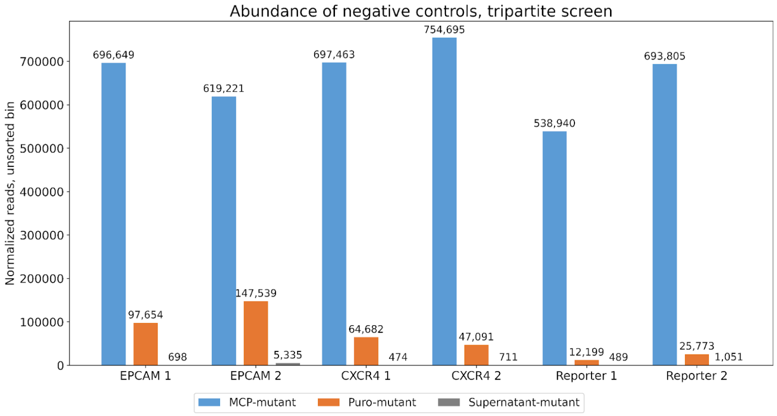

Supplementary Figure 37. *Abundance of negative control barcodes (MCP-mutant – blue, Puro-mutant – orange, Supernatant-mutant – gray) in tripartite screens.*

Supplementary Note 2. Establishment of protein folders as inert positional placeholders

Protein folders (PFs) SG_GB1 (A23), HS_MBP (A24), and EC_TRXA (A25) were included as inputs into our combinatorial libraries (along with the 22 activation domains (ADs)) to promote the stability of each fusion and to serve as negative controls. However, upon testing, A24 was found to be consistently detrimental to activator performance and was subsequently excluded from all analyses utilizing PFs.

In order to establish the remaining PFs, A23 and A25, as inert with respect to toxicity and activation, we performed the following analyses using both screen-derived toxicity scores (averaged across *EPCAM* and *CXCR4*) and screen-derived activation scores (for activation, analyses were performed on all three target genes):

We calculated the Pearson correlation between each AD's score in the single-domain screen with the median of bipartite screen scores for all instances of one copy of the corresponding AD paired with either of two inert PFs in the remaining position. We also calculated the Pearson correlations between each AD's score in the single-domain screen and the median of tripartite screen scores for all instances of one copy of the corresponding AD paired with either of two inert PFs in the remaining positions.

The high correlations yielded by these analyses for both toxicity and activation (**Supplementary Figures 18a-b**) demonstrate that PFs either exert the same effects on all ADs or negligible effects on all ADs. As we consider the latter explanation to be more likely, we used these data as justification for the use of PFs as inert placeholders in downstream toxicity and activation analyses. Specifically, AD1-PF and AD1-PF-PF are used as surrogates for one copy of AD1 within copy number analyses for the bipartite and tripartite screens, respectively. AD1-AD2-PF is a surrogate for AD1-AD2 within order analyses for the tripartite screen.

Supplementary Note 3. Assessment of lentiviral recombination

To evaluate the potential impact of lentiviral recombination on the performance of our high-throughput screen, we performed a dedicated control experiment. We designed two barcoded lentiviral MCP activator constructs: one containing a functional blasticidin resistance gene and one containing a non-functional version of the gene (**Supplementary Figure 38**). Each construct was uniquely barcoded and flanked by shared priming sites. The two constructs were mixed at equal molar ratios and packaged into lentivirus following the same protocol used for our screen.

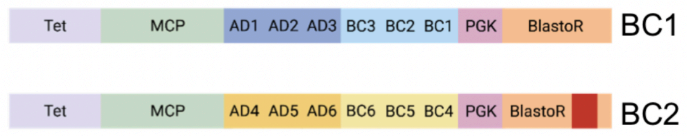

Supplementary Figure 38. *Schematic of constructs used to assess lentiviral recombination. The top construct contains an intact blasticidin resistance gene; the bottom construct contains a non-functional version. Each construct is uniquely barcoded (BC1 and BC2) and shares common priming sites for barcode amplification.*

The resulting viral mixture was transduced into HEK293T cells – the same cell type used for screening – at a range of multiplicities of infection (MOIs). Cells were then cultured in 5 µg/mL blasticidin for four passages to select for cells harboring functional blasticidin resistance. After selection, genomic DNA was extracted, and the barcode region was PCR-amplified using primers targeting the shared flanking sequences. Amplicons were sequenced to quantify the relative abundance of barcodes corresponding to the intact and mutant blasticidin resistance constructs.

We hypothesized that any barcode swapping caused by lentiviral recombination or packaging artifacts would result in retention of barcodes associated with the non-functional blasticidin resistance construct in blasticidin-resistant cells. At low MOI (1 µL virus condition), we observed that barcode swapping was infrequent – occurring in less than 5% of reads (**Supplementary Figure 39**). As expected, higher MOIs showed increased frequencies of the mutant blasticidin resistance barcode, likely reflecting cells that had received multiple viral integrations (e.g., one with a functional and one with a mutant construct).

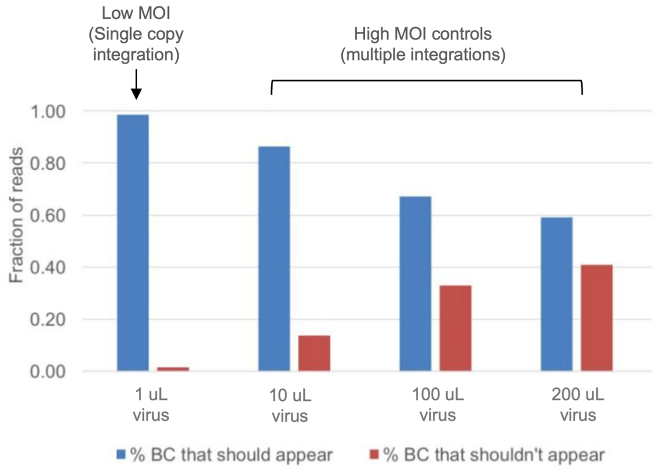

Supplementary Figure 39. *Quantification of barcode presence across varying MOIs. At low MOI (1 µL virus), the vast majority of reads correspond to the functional construct (blue). At higher MOIs, barcodes from the non-functional construct (red) increase in frequency, consistent with multiple integrations.*

These results indicate that barcode swapping occurred rarely under the low-MOI conditions used in our screen, and that our screening platform is robust to lentiviral recombination artifacts.

Supplementary Tables

Supplementary Table 1. A-IDs of combinatorially assembled domains

A-ID	Domain name
A01	HHV1_VP16x2
A02	CercAHV2_VP16x2
A03	HS_MLLx3
A04	JMSFV_BEL1
A05	FBAHV1_VP64
A06	CercAHV2_VP16
A07	SFV3_BEL1
A08	HHV8_VIRF2
A09	SaHV1_VP16
A10	HS_C3ORF62
A11	BAHV1_ICP4-11
A12	HHV8_ORF50
A13	HS_FAM22F
A14	HS_MASTR
A15	CervAHV2_ICP4
A16	HAVF41_E1A
A17	HS_E2F2
A18	HS_CITED2x4
A19	HHV1_VP64
A20	HS_P65
A21	EBV_RTA
A22	HS_HSF1
A23	SG_GB1
A24	EC_MBP
A25	EC_TRXA

Supplementary Table 2. Guide RNA sequences used in this study

Organism	Target gene	Location	Sequence
Homo sapiens	*NEUROD1*	-52	AGGGGAGCGGTTGTCGGAGG
Homo sapiens	*TTN*	-169	CCTTGGTGAAGTCTCCTTTG
Homo sapiens	*HBG1*	-100	CTTGACCAATAGCCTTGACA
Homo sapiens	*RHOXF2*	-44	ACGCGTGCTCTCCCTCATC
Homo sapiens	*CXCR4*	-116	GCAGACGCGAGGAAGGAGGGCGC
Homo sapiens	*CXCR4*	-192	CCGACCACCCGCAAACAGCA
Homo sapiens	*CXCR4*	-193	GCCTCTGGGAGGTCCTGTCCGGCTC
Homo sapiens	*EPCAM*	-108	TCACTCCCCCAACTCCCGGG
Homo sapiens	*EPCAM*	-168	GCGGCCCTCTCCACAGGTGT
Homo sapiens	*EPCAM*	-84	GACTCATCAACGAGCACCAG
Homo sapiens	*EGFR*	-103	AGGGAGGAGAACCAGCAGCG
Homo sapiens	*CD2*	-18	GAGGCACGTGGTTAAGCTCT
Homo sapiens	*CD45*	-39	TGCTAGCTGCATGAACTGCT
Mus musculus	*ttn*	-143	AATTTAGCACTGCCAATCAG
Mus musculus	*hbb-bh1*	-148	AGAGAGTCTGGGCAAGACAG

Supplementary Table 3. Filtering parameters used for NGS analyses

Screen	Cutoff to be considered UMI outlier	Bin UMI on N bases to create group	Total UMI groups	Discard if any sorted condition equals	Discard if sum of UMI group reads across sorted conditions is less than
Single-domain	0.05% of total population	3	64	NA	4
Bipartite	0.05% of total population	2	16	NA	20
Tripartite	1% of total population	0	0	0	493

Supplementary Table 4. qPCR primer sequences used in this study

Organism	Gene	Forward qPCR primer sequence	Reverse qPCR primer sequence
Homo sapiens	*ACTC1*	ATGTGTGACGACGAGGAGAC	CGGACAATTTCACGTTCAGCA
Homo sapiens	*ASCL1*	CGCGGCCAACAAGAAGATG	CGACGAGTAGGATGAGACCG
Homo sapiens	*HBG1*	AGATGCCACAAAGCACCTG	CTGCAGTCACCATCTTCTGC
Homo sapiens	*IL1B*	CCACAGACCTTCCAGGAGAATG	GTGCAGTTCAGTGATCGTACAGG
Homo sapiens	*IL1R2*	GGCTATTACCGCTGTGTCCTGA	GAGAAGCTGATATGGTCTTGAGG
Homo sapiens	*IL1RN*	GGAATCCATGGAGGGAAGAT	TGTTCTCGCTCAGGTCAGTG
Homo sapiens	*LIN28A*	CCAGTGGATGTCTTTGTGCACC	GTGACACGGATGGATTCCAGAC
Homo sapiens	*MIAT*	TGGCTGGGGTTTGAACCTTT	AGGAAGCTGTTCCAGACTGC
Homo sapiens	*NEUROD1*	GGATGACGATCAAAAGCCCAA	GCGTCTTAGAATAGCAAGGCA
Homo sapiens	*RHOXF2*	GGCAAGAAGCATGAATGTGA	TGTCTCCTCCATTTGGCTCT
Homo sapiens	*TTN*	TGTTGCCACTGGTGCTAAAG	ACAGCAGTCTTCTCCGCTTC
Homo sapiens	*ZFP42*	CGCAATCGCTTGTCCTCAGAGT	GCTCTCAACGAACGCTTTCCCA
Mus musculus	*hbb-bh1*	CACAGCTGAGGAGAAGGCAGC	GCACTTTCTTGCCATGGGCTC
Mus musculus	*Ins2*	GGCTTCTTCTACACACCCAT	CCAAGGTCTGAAGGTCACCT
Mus musculus	*Itgav*	GTGTGAGGAACTGGTCGCCTAT	CCGTTCTCTGGTCCAACCGATA
Mus musculus	*Mef2d*	CTTCGCGTAACCGAGGATT	TGTGCTCATGAATGTCTGGG
Mus musculus	*Neurog2*	CAAAGTCGCCCAGCCGAGA	GATTTGACGAACATCCTACGC
Mus musculus	*Sim1*	CACTATCTCGGACAACAGGAAGG	CTGGCTGTCATGGTCAGATTCC
Mus musculus	*Slc7a11*	AGGGCATACTCCAGAACACG	GGACCAAAGACCTCCAGAATG
Mus musculus	*ttn*	GACACCACAAGGTGCAAAGTC	CCCACTGTTCTTGACCGTATCT

Supplementary Figures

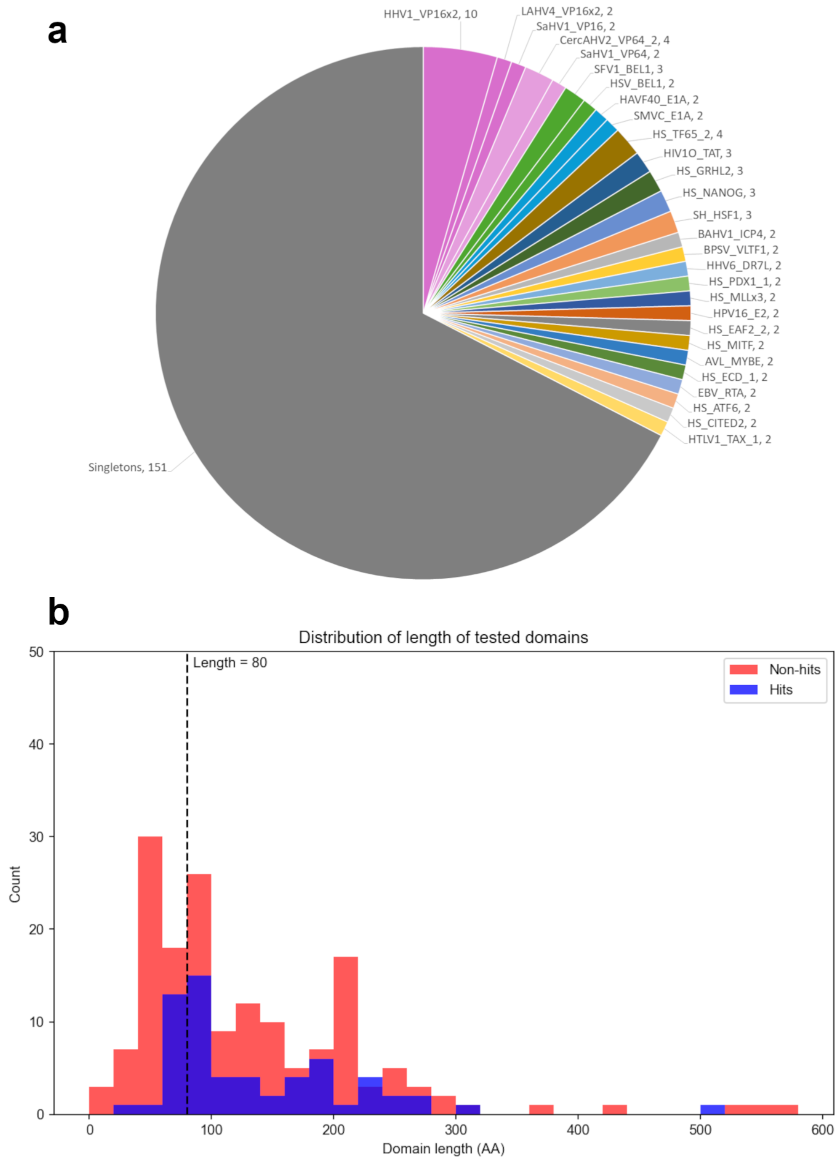

Supplementary Figure 1. Diversity of individually tested domains. (a) All 230 individually tested parts were clustered at 50% sequence identity using uclust. Families of related activators that were clustered into multiple sets were grouped and colored similarly. Clusters are labeled with their centroid member's name followed by the size of the cluster. (b) Distribution of lengths of 230 tested domains, separated between hits and non-hits. Line at 80 amino acids shows the length associated with commonly used 80-AA tiling methods. Each domain was tested in duplicate (n = 2 biological replicates). Source data are provided as a Source Data file (Source Data.zip).

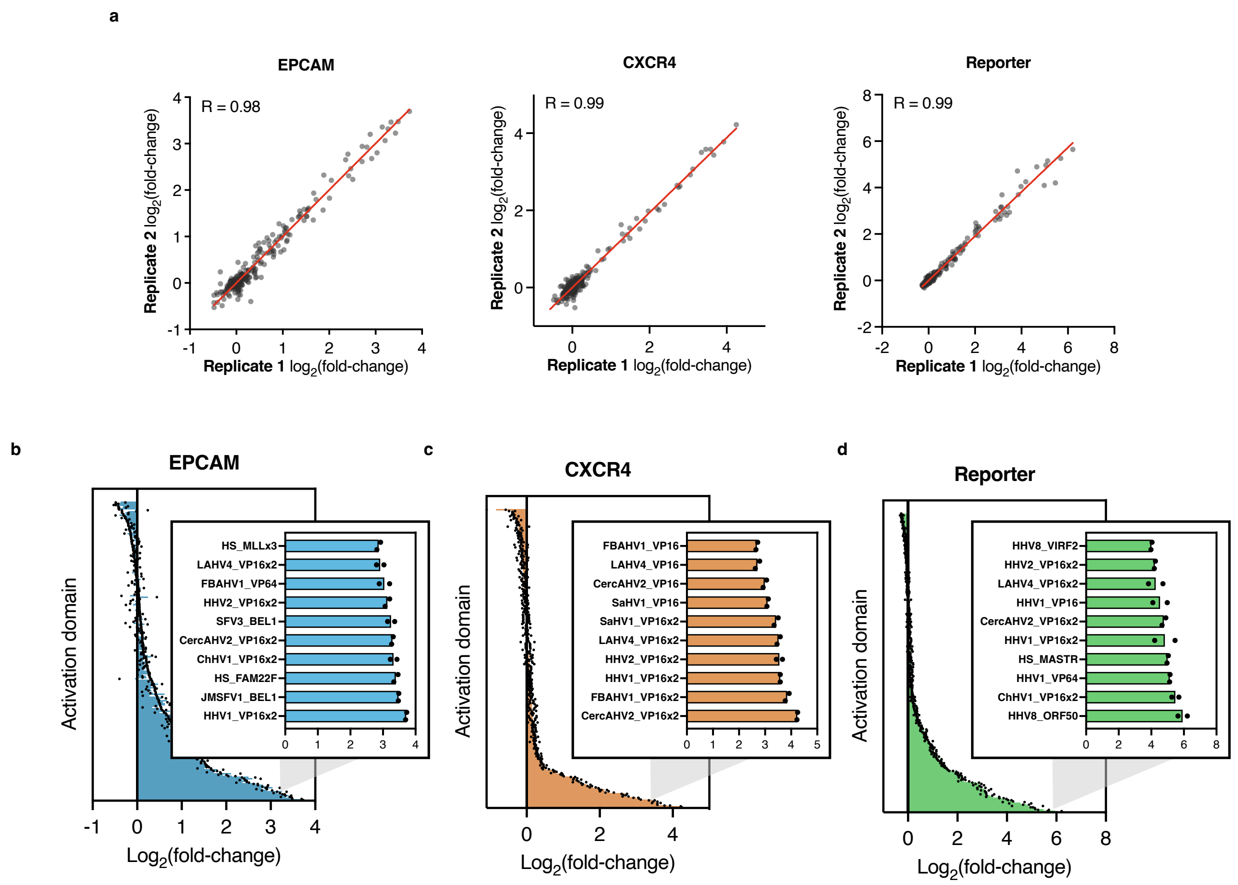

Supplementary Figure 2. Performance of activators across multiple target genes in biological duplicate. (a) Correlation between $\log_2$(fold-changes) in *EPCAM*, *CXCR4*, and synthetic reporter expression for each activator across transfection replicates. Correlations were calculated using Pearson correlation coefficient (*R*). (b-d) Log_2(fold-change) in *EPCAM*, *CXCR4*, and synthetic reporter expression, respectively, following targeting by 230 dCas9 fusions. Inset shows top 10 activators. Data are normalized to a dCas9-only negative control. Plots include VP16 variants. Black dots depict results of two independent transfections. For all panels, *n* = 2 biological replicates. Source data are provided as a Source Data file (Source Data.zip).

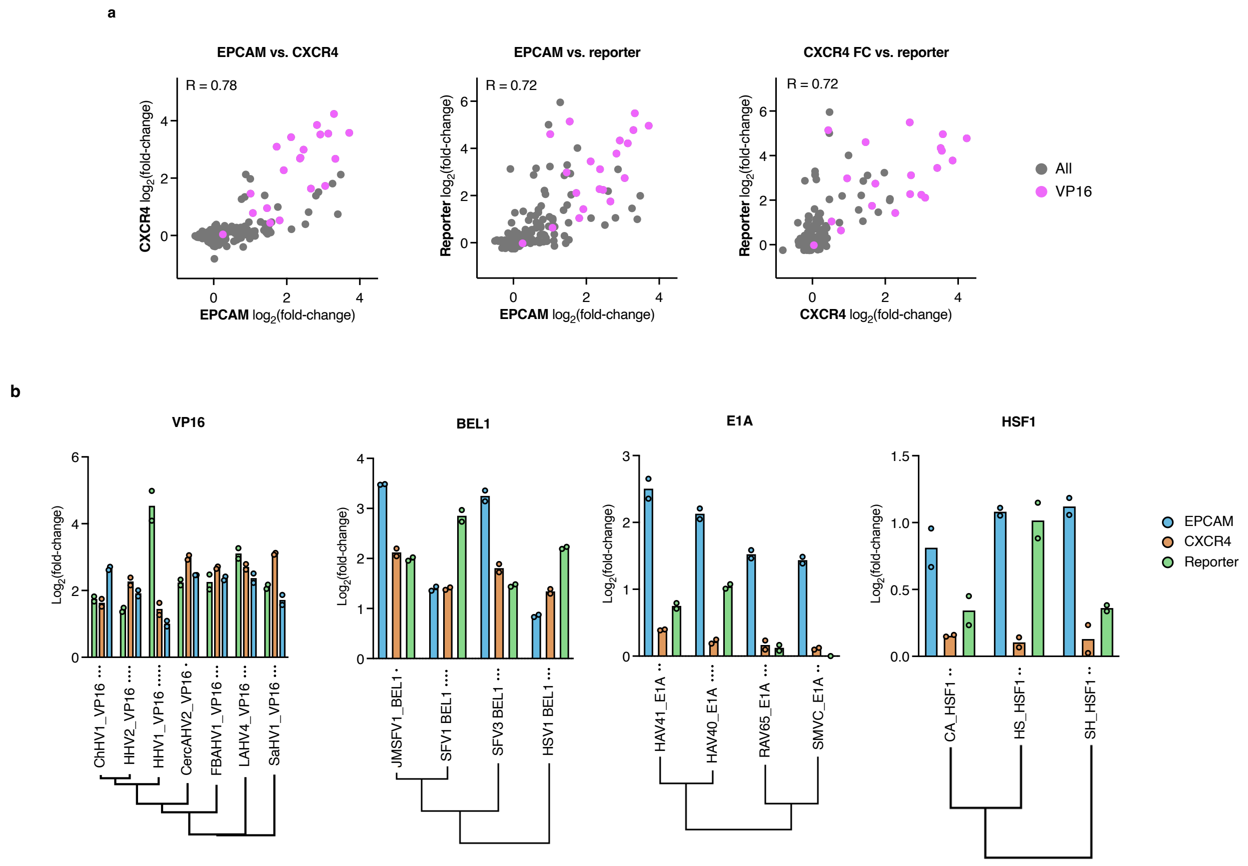

Supplementary Figure 3. AD homologs exhibit similar activation profiles. (a) Correlation between $\log_2$(fold-changes) in target expression for 230 dCas9-fusions targeting *EPCAM* and *CXCR4*, *EPCAM* and the synthetic reporter, and *CXCR4* and the synthetic reporter. VP16 variants (pink) are shown. Data are shown as the mean (n = 2 independent transfections). Correlations were calculated using Pearson correlation coefficient (R). (b) Log_2(fold-changes) in target expression for VP16, BEL1, E1A, and HSF1 homologs against *EPCAM* (blue), *CXCR4* (orange), and the synthetic reporter (green). Dendrograms below each graph are provided to illustrate the phylogenetic relationships among homologs. Dots depict the performance of activators based on two independent transfections (n = 2 biological replicates). For all panels, data are normalized to a dCas9 negative control plasmid. Source data are provided as a Source Data file (Source Data.zip).

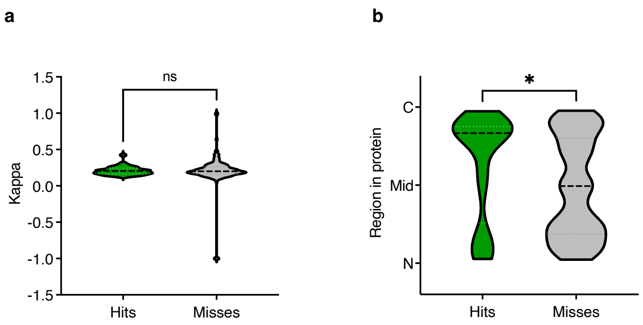

Supplementary Figure 4. Examination of biochemical traits underlying activator function. (a) Kappa (degree of mixing between positively and negatively charged amino acids, lower = greater mixing) for hits (green) and misses (gray). (b) The center of the region from which each activator is derived in its native protein for hits (green) and misses (gray). Native protein length (y-axis) was first normalized to represent each point as a fraction of the total protein length to facilitate comparisons across protein sequences with variable lengths. N, Mid, and C show the N-terminus, middle, and C-terminus, respectively, of activators' native proteins. Violins were truncated at the y-axis values of N and C to reflect the biological constraints of protein length and to exclude values that imply locations beyond the physical ends of the protein. For all panels, the significance of the difference in values between hits and misses was assessed via unpaired two-sided t-test (n = 230 domains tested in biological duplicate). For * and ns, p = 0.0231 and not significant, respectively. Source data are provided as a Source Data file (Source Data.zip).

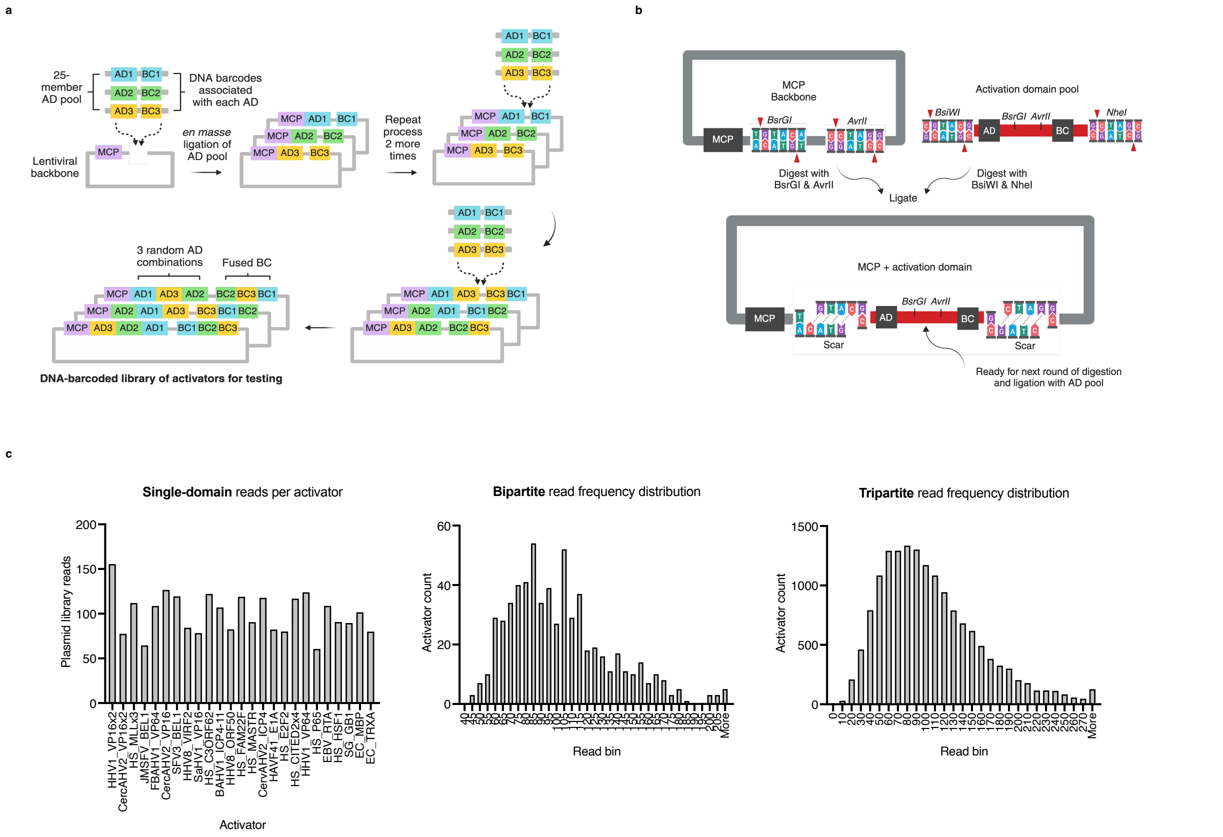

Supplementary Figure 5. High-throughput combinatorial cloning generates thousands of activation domain combinations. (a) Schematic depicting combinatorial assembly of ADs downstream of MCP. An insert pool consisting of 25 parts (ADs and protein folding domains), each appended with a 3' barcode, is cloned in one reaction downstream of MCP. This step generates a series of vectors each containing one insert and an adjacent barcode. This pool is then redigested, and the same set of 25 inserts are reintroduced. By repeating this cloning process a total of two to three times, a set of constructs containing all possible two- or three-way combinations of the 25 parts fused downstream of MCP are produced. A distinct feature of this construction process is that all generated vectors also contain a DNA barcode that can be used to decipher their identities (*Created in BioRender. Giddins, M. (2025) https://BioRender.com/oeyf739*). (b) Schematic depicting restriction cloning strategy for iterative AD insertions downstream of MCP. The MCP backbone harbors BsrGI and AvrII sites downstream of MCP. Each part in the pool harbors a short downstream barcode, along with a BsiWI site upstream of each part and a NheI site downstream of the barcode. BsrGI and AvrII sites also sit in between each AD and barcode. Digestion of the MCP backbone with BsrGI and AvrII and the activation domain insert pool with BsiWI and NheI create compatible overhangs, which can be ligated together. The ligated constructs form scars between the BsrGI-BsiWI and AvrII-NheI fused sites, which cannot be digested by either of the original enzymes. The BsrGI and AvrII sites in between the previously inserted AD and its barcode allow for another round of digestion of the pool with BsrGI and AvrII, enabling the entire cloning process to be repeated (*Created in BioRender. Giddins, M. (2025) https://BioRender.com/b0mamqf*). (c) Distribution of members within each library. NGS reads counted for each member of the single-domain, bipartite and tripartite plasmid libraries are presented. For bipartite and tripartite libraries, reads are binned by sets of 10, and all activators that produced over 205 or 270 reads, respectively, were placed into overflow (more) bins. Source data are provided as a Source Data file (Source Data.zip).

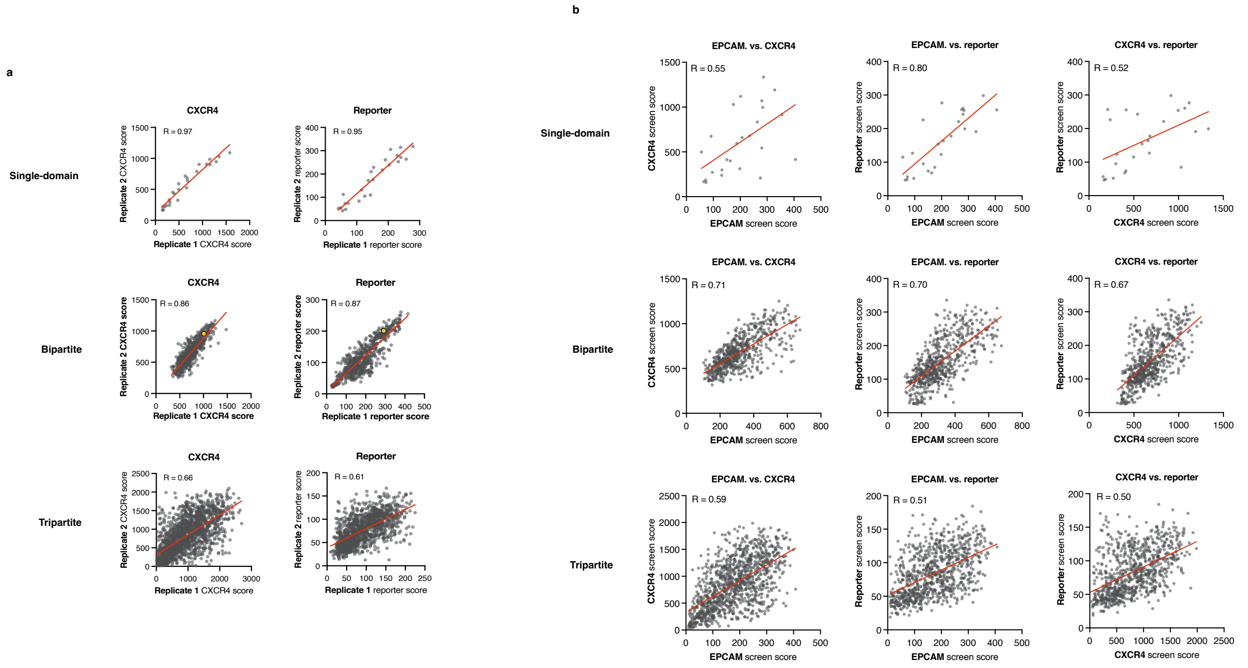

Supplementary Figure 6. Correlations between screen-generated activator scores across biological replicates and across targets. (a) Correlations between screen scores across two biological replicates for single-domain, bipartite, and tripartite libraries targeted to *CXCR4* or the reporter. Yellow dot (bipartite panel, middle) indicates performance of MCP-P65-HSF1 (MCP component of SAM). (b) Correlation between screen scores against different targets for the single-domain, bipartite, and tripartite libraries. Data are shown as the mean (*n* = 2 independent screen replicates). For all panels, correlations were calculated using Pearson correlation coefficient (*R*). Source data are provided as a Source Data file (Source Data.zip).

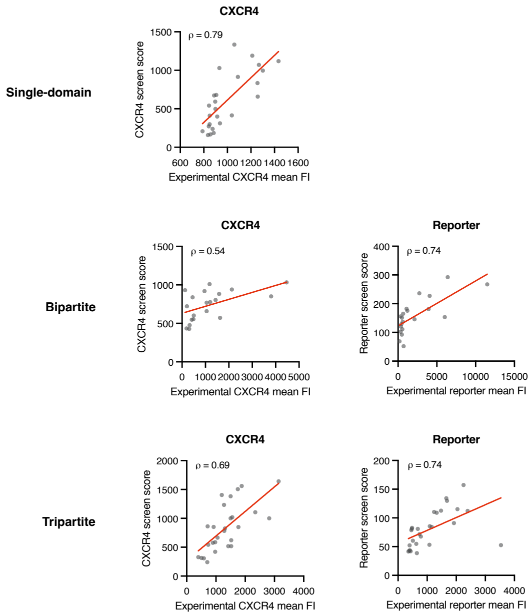

Supplementary Figure 7. Experimental validation of screening data to evaluate the efficacy of our high-throughput approach. Correlations between activator screen scores and target expression derived from individual testing are shown for *CXCR4* (single-domain, bipartite, and tripartite screens) and the synthetic reporter (bipartite and tripartite screens). Experimental data (x-axis) are presented as the mean (n = 2 independent transduction replicates). Screening data (y-axis) are shown as the mean (n = 2 independent screen replicates). Correlations were calculated using Spearman correlation coefficient (ρ). Source data are provided as a Source Data file (Source Data.zip).

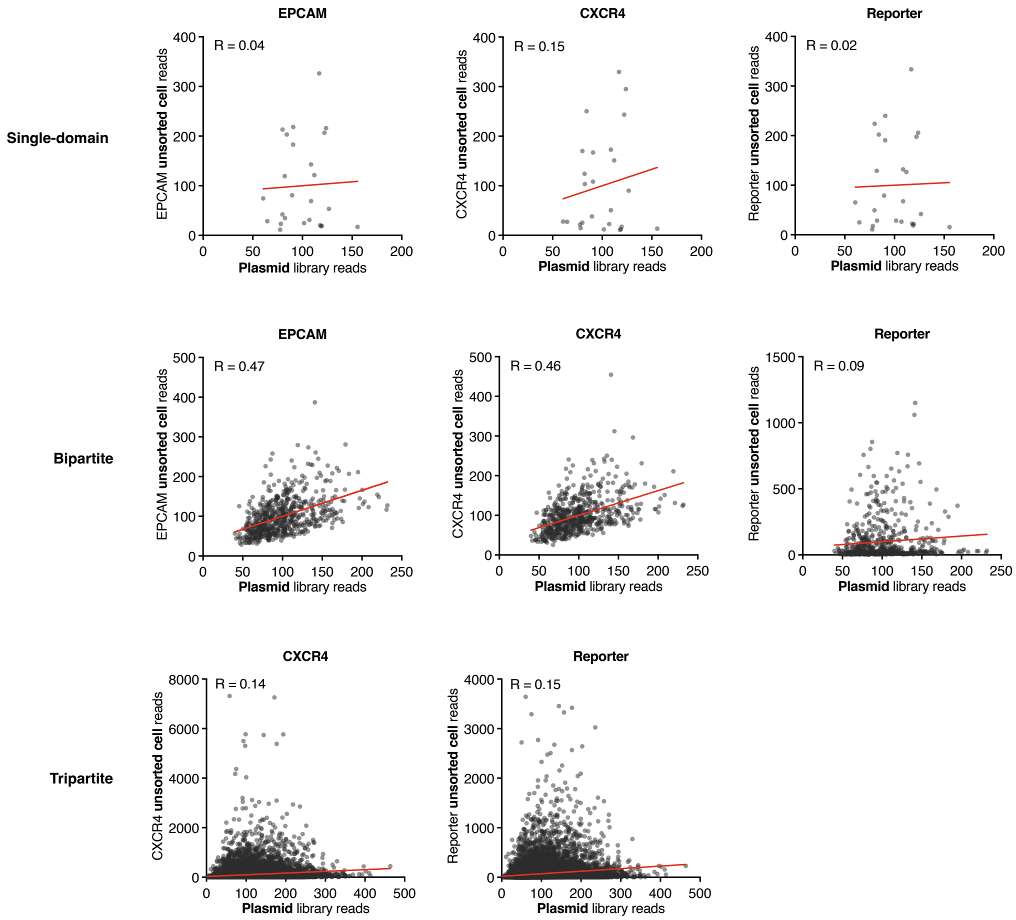

Supplementary Figure 8. Library member abundances in cells exhibit weak or no correlations with library member abundances in plasmid libraries. Correlations between numbers of plasmid library reads and numbers of *EPCAM*, *CXCR4*, and reporter unsorted cell reads for various libraries are shown. Cell data (y-axes) are presented as the mean (*n* = 2 independent screen replicates). Correlations were calculated using Pearson correlation coefficient (*R*). Source data are provided as a Source Data file (Source Data.zip).

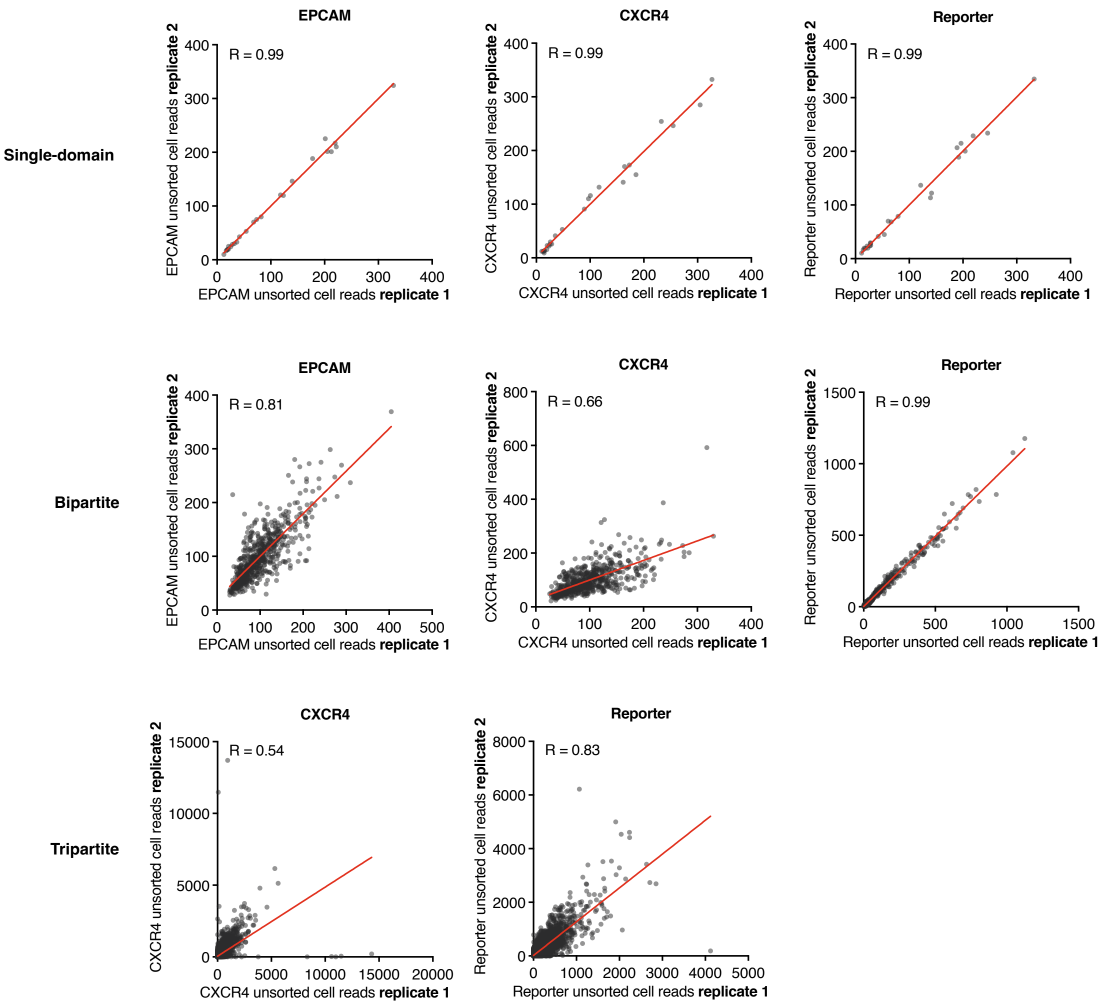

Supplementary Figure 9. Library member abundances are similar across biological replicates in cells. The correlation between numbers of reads for two unsorted cell biological replicates within *EPCAM*- (single-domain and bipartite), *CXCR4*- (single-domain, bipartite, and tripartite), and synthetic reporter- (single-domain, bipartite, and tripartite) targeting screens are shown. For *EPCAM*- and *CXCR4*-targeting screens, data shown represent cells harvested 10, 11, and 11 days post-infection for the single-domain, bipartite, and tripartite screens, respectively, following three passages in 0.5 μg/mL puromycin selection. For the reporter-targeting screen, data shown represent cells harvested 13, 14, and 14 days post-infection for the single-domain, bipartite, and tripartite screens, respectively. Correlations were calculated using Pearson correlation coefficient (*R*). Source data are provided as a Source Data file (Source Data.zip).

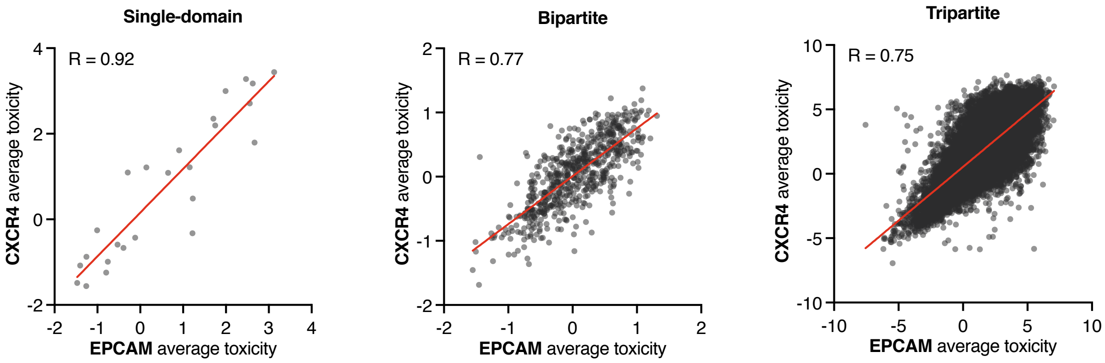

Supplementary Figure 10. Activator toxicity does not depend on the target gene. The correlation between *EPCAM*-specific and *CXCR4*-specific toxicity scores for the single-domain, bipartite, and tripartite screens are shown. Toxicity scores depict the $-\log_2$(number of reads in the final unsorted cell library/number of reads in the plasmid library). Data were derived from two independent screen replicates for each target (n = 2 biological replicates). Correlations were calculated using Pearson correlation coefficient (R). Lower (more negative) values correspond to lower toxicity. Source data are provided as a Source Data file (Source Data.zip).

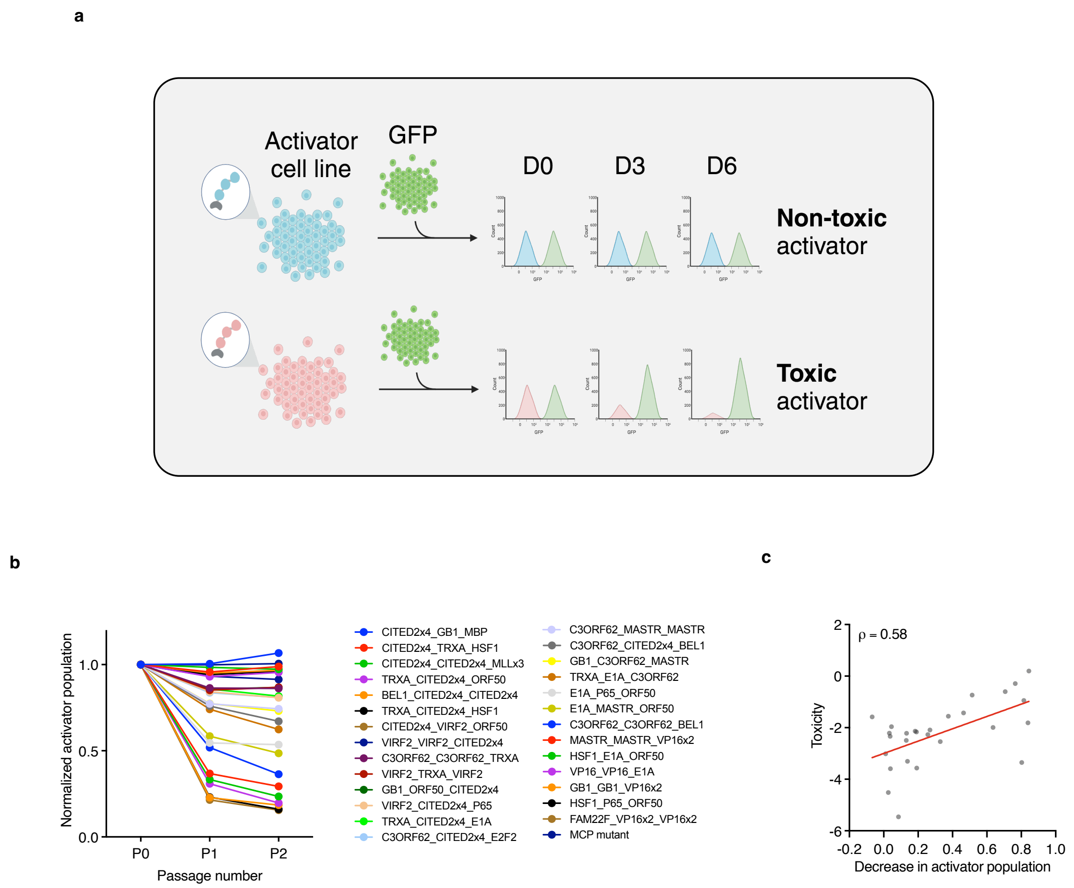

Supplementary Figure 11. Experimental validation of screen-derived toxicity scores. (a) Schematic for experimentally evaluating activator toxicity. Activators randomly chosen from the tripartite library were made into stable cell lines in dCas9- and *CXCR4* gRNA-expressing HEK293T cells. Activator-expressing cell lines were each mixed at a 1:1 ratio with HEK293T cells stably expressing MCP fused to GFP (MCP-GFP). Every 72 hours, cell mixes were split 1:8, and GFP expression was quantified using flow cytometry. Non-toxic activators (blue) are expected to show smaller reductions in population size (as evaluated by the fraction of the non-GFP population, non-green) than toxic activators (pink) over time (*Created in BioRender. Giddins, M. (2025) https://BioRender.com/cgc9g54*). (b) Growth of tripartite clones isolated from the tripartite library, after being mixed 1:1 with an MCP-GFP control-expressing line and passaged at a 1:8 ratio twice. Activator population sizes (y-axis, non-GFP populations) were normalized to their baseline levels at the start of the experiment. The abundance of each activator at each of two passages, P1 and P2, on the x-axis, are shown. A construct containing a mutated MCP protein (dark blue), in which the sixth amino acid in the sequence, glutamine, was changed to a TAG stop codon, was included as a control. Data are shown as the mean of two independent transduction replicates. (c) Correlation between the reduction in population size of each individually tested activator over the passaging experiment (x-axis) and its screen-produced toxicity score (y-axis). Correlations were calculated using Spearman correlation coefficient (ρ). Lower (more negative) values correspond to lower toxicity. Source data are provided as a Source Data file (Source Data.zip).

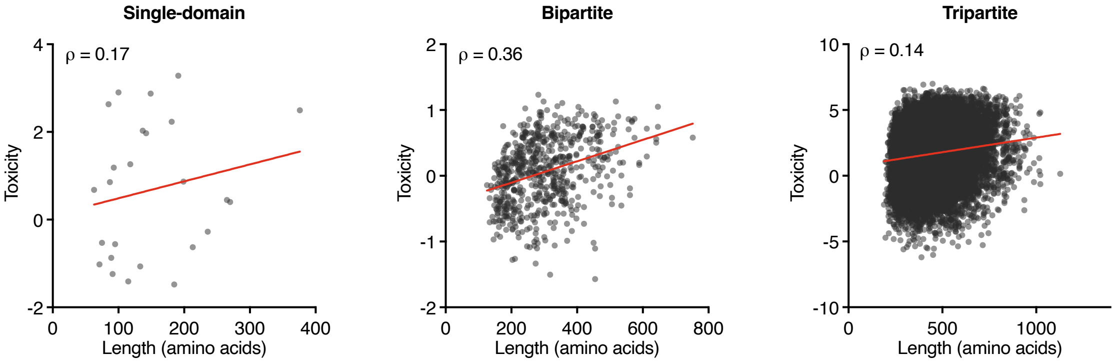

Supplementary Figure 12. Activator toxicity correlates weakly with total construct length. Correlations between the total length of each AD or AD combination and its toxicity score for single-domain, bipartite, and tripartite screens are shown. Correlations were calculated using Spearman correlation coefficient (ρ). Lower (more negative) values correspond to lower toxicity. Source data are provided as a Source Data file (Source Data.zip).

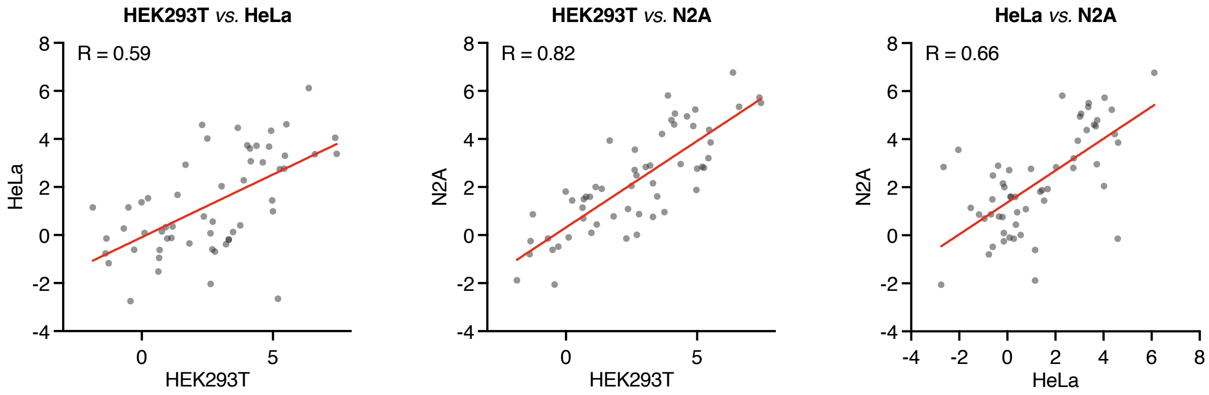

Supplementary Figure 13. Activator toxicity does not depend on cell type. The correlations between activator toxicity scores across cell types following delivery of a 56-member mini-library into HEK293T, HeLa, and N2A cells are shown. Toxicity scores depict the $-\log_2$(number of reads in the final unsorted cell library/number of reads in the plasmid library). Data were derived from two independent transduction replicates for each cell type. Correlations were calculated using Pearson correlation coefficient (*R*). Lower (more negative) values correspond to lower toxicity. Source data are provided as a Source Data file (Source Data.zip).

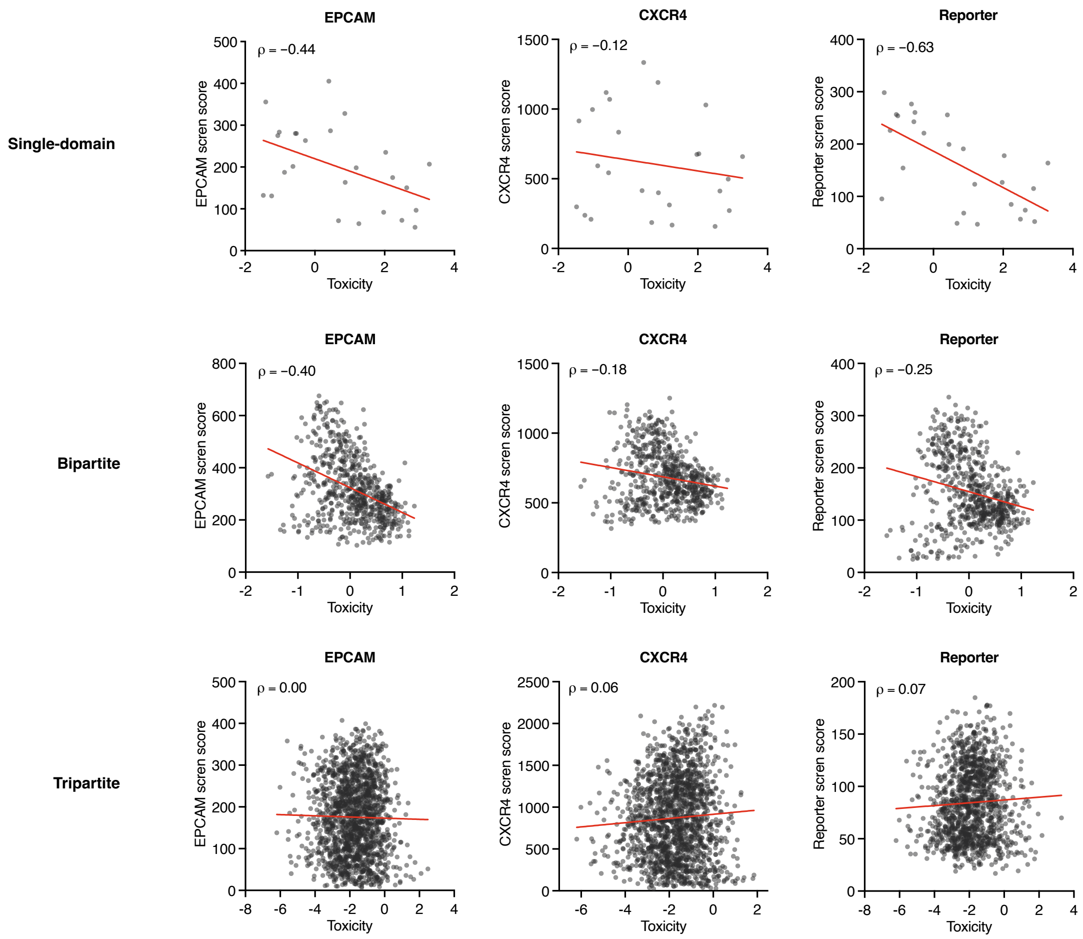

Supplementary Figure 14. Activator toxicity does not correlate with activation. The correlations between toxicity scores and screen-derived activation scores for single-domain, bipartite, and tripartite libraries across the various target genes are shown. Correlations were calculated using Spearman correlation coefficient (ρ). For all panels, lower (more negative) values correspond to lower toxicity. Source data are provided as a Source Data file (Source Data.zip).

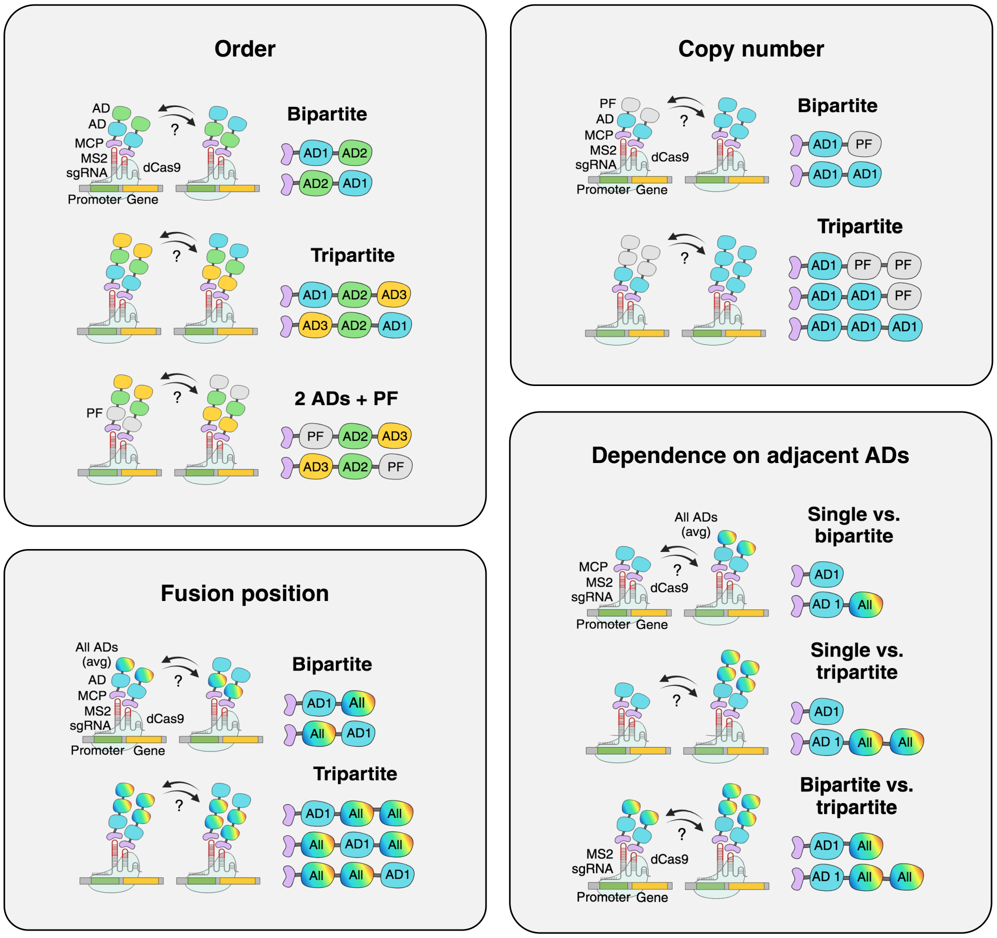

Supplementary Figure 15. Schematics depicting four properties of assembled activators that were explored using screening data: the effect of order, fusion position, copy number, and adjacent ADs on activity (*Created in BioRender. Giddins, M. (2025) https://BioRender.com/rea92o2*).

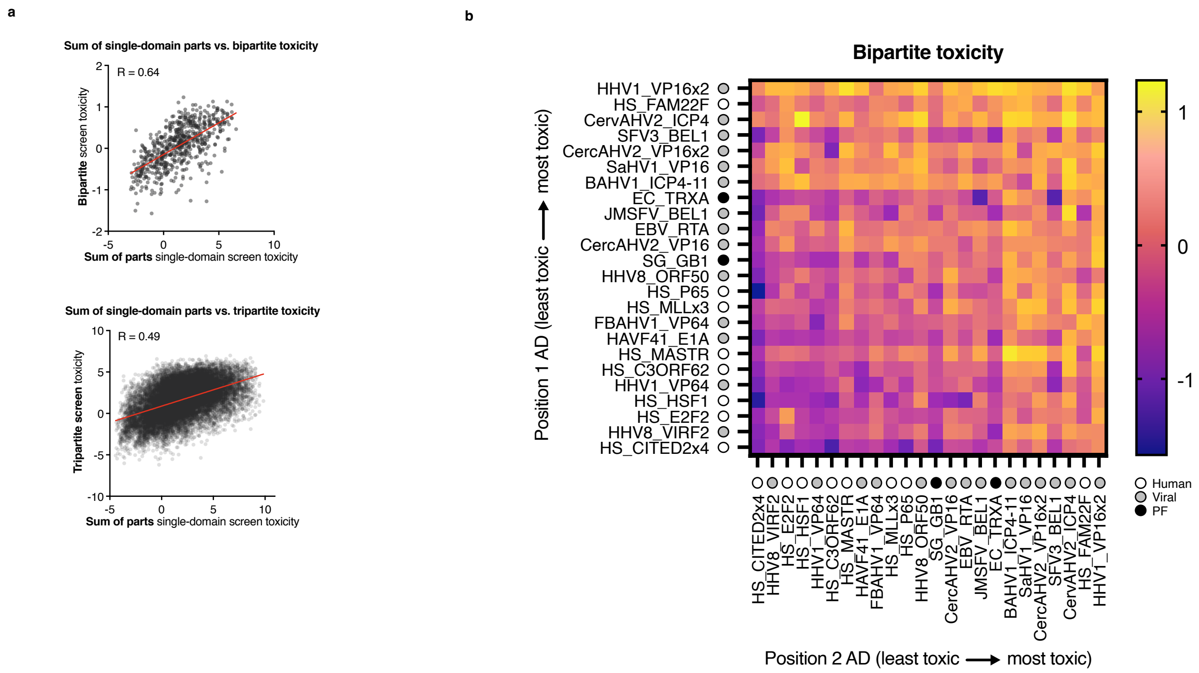

Supplementary Figure 16. The toxicity of multi-partite constructs can be predicted by summing the toxic effects of their individual parts. (a) For each bipartite or tripartite activator, toxicity (y-axis) was compared to the sum of the toxicities of its individual domains as determined from the single-domain screen (x-axis). Correlations were calculated using Pearson correlation coefficient (*R*). (b) Bipartite toxicity scores produced by each of 25 ADs in position one, the position closest to MCP (y-axis), upon pairing with each of 25 ADs in position two, the position most distal to MCP (x-axis). ADs are ordered from least to most toxic within the single-domain screen, where purple blocks = lower toxicity, yellow blocks = higher toxicity. Each activator is annotated based on the species it derives from or its functionality as a human AD (white dots), viral AD (gray dots), or PF (black dots). For all panels, lower (more negative) values correspond to lower toxicity. Source data are provided as a Source Data file (Source Data.zip).

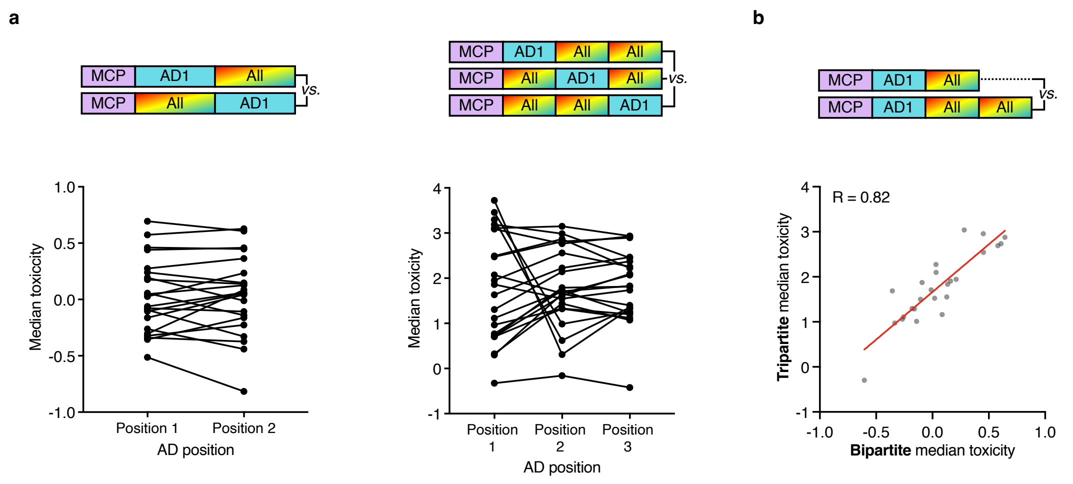

Supplementary Figure 17. Domain toxicity does not depend on fusion position or adjacent elements. (a) Median toxicity scores produced by ADs in all possible fusion positions. Values were produced by calculating the median of toxicity scores of all constructs containing a given AD at the listed position. (b) Correlation between median toxicity scores of individual ADs within bipartite and tripartite screens. Values were produced by calculating the median of toxicity scores of all constructs containing a given AD within a given screen. Correlations were calculated using Pearson correlation coefficient (*R*). For all panels, lower (more negative) values correspond to lower toxicity. Source data are provided as a Source Data file (Source Data.zip).

a

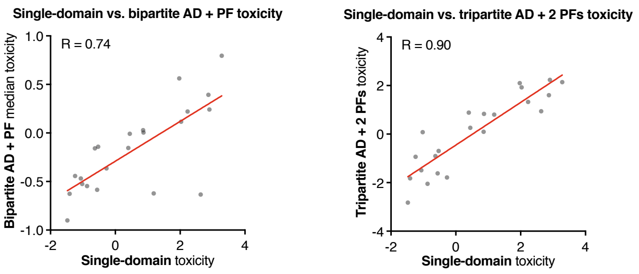

b

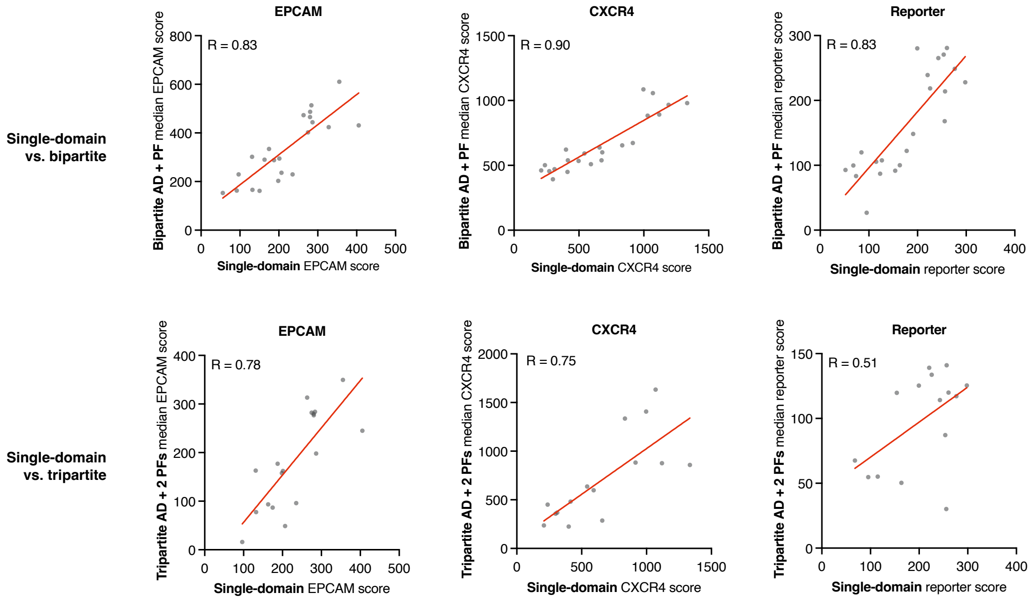

Supplementary Figure 18. Protein folders do not affect activator activation or toxicity. (a) (Left) correlation between each AD's single-domain screen toxicity score and the median bipartite screen toxicity scores for one copy of that AD paired with a PF. (Right) correlation between each AD's single-domain screen toxicity score and the median of tripartite screen toxicity scores for one copy of that AD paired with PFs occupying the remaining positions. Lower (more negative) values correspond to lower toxicity. (b) (Top) correlations between each AD's single-domain screen score and the median bipartite screen scores for one copy of that AD paired with a PF within the *EPCAM*, *CXCR4*, and reporter screens. (Bottom) correlations between each AD's single-domain screen score and the median tripartite screen scores for one copy of that AD paired with PFs occupying the remaining positions within the *EPCAM*, *CXCR4*, and reporter screens. Data are shown as the mean (n = 2 screen replicates). For all panels, correlations were calculated using Pearson correlation coefficient (R). Source data are provided as a Source Data file (Source Data.zip).

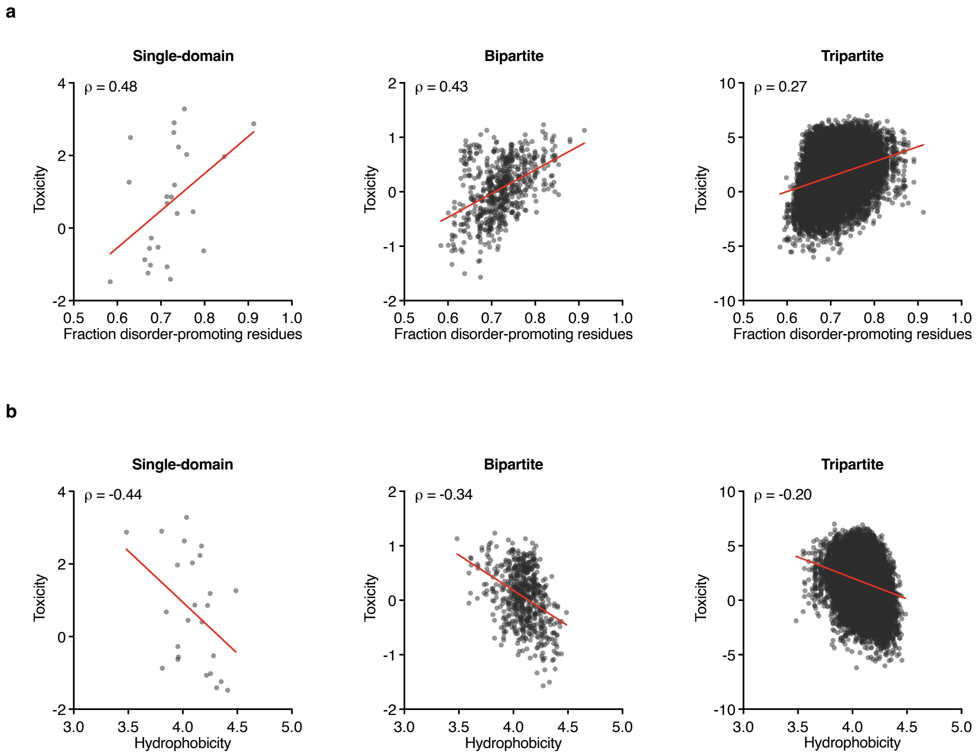

Supplementary Figure 19. Fraction of disorder-promoting residues and hydrophobicity show positive and negative correlations, respectively, with activator toxicity. (a) Correlation between fraction of disorder-promoting residues and toxicity for activators in the single-domain, bipartite, and tripartite screens. (b) Correlation between hydrophobicity and toxicity for activators in the single-domain, bipartite, and tripartite screens. Fraction of disorder-promoting residues (a) and hydrophobicity (b) were calculated for the entire fusion downstream of MCP. All correlations were calculated using Spearman correlation coefficient (ρ). For all panels, lower (more negative) values correspond to lower toxicity. Source data are provided as a Source Data file (Source Data.zip).

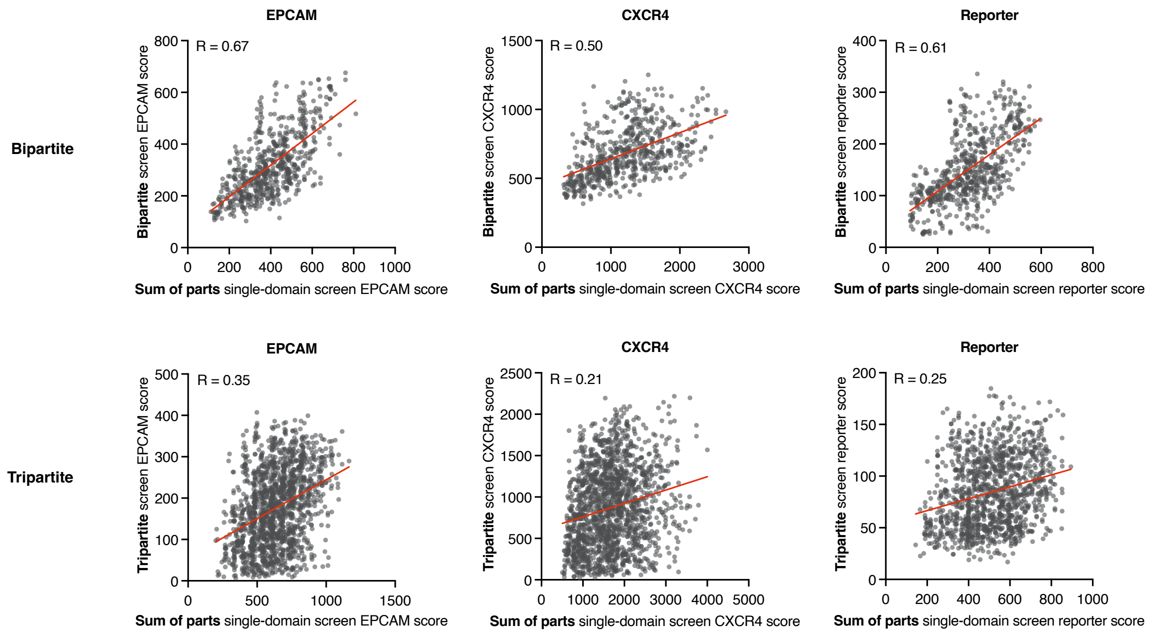

Supplementary Figure 20. The strength of bipartite but not tripartite tools can be predicted by summing the activation potentials of their individual domains. For each bipartite or tripartite activator within *EPCAM*-, *CXCR4*-, and reporter-targeting screens, activation (y-axis) was compared to the sum of activation scores of its individual domains as determined from the corresponding single-domain screen (x-axis). Data are shown as the mean (n = 2 independent screen replicates). Correlations were calculated using Pearson correlation coefficient (R). Source data are provided as a Source Data file (Source Data.zip).

a

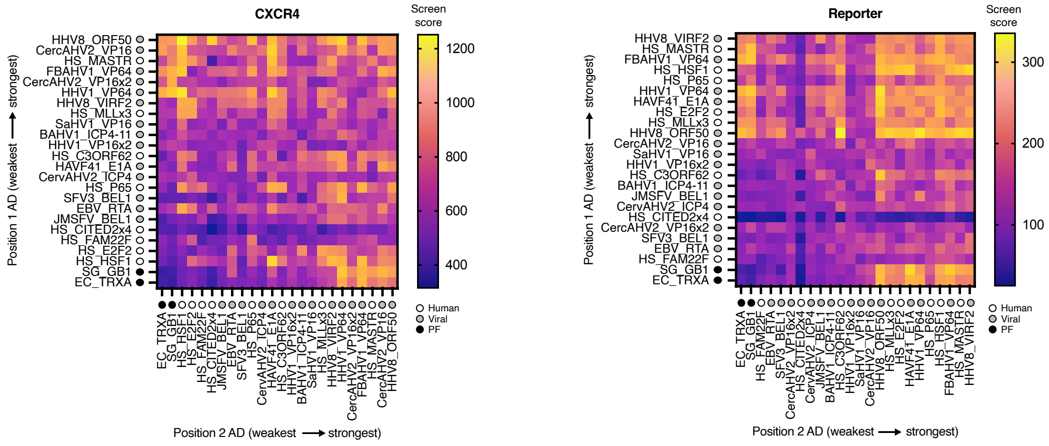

b

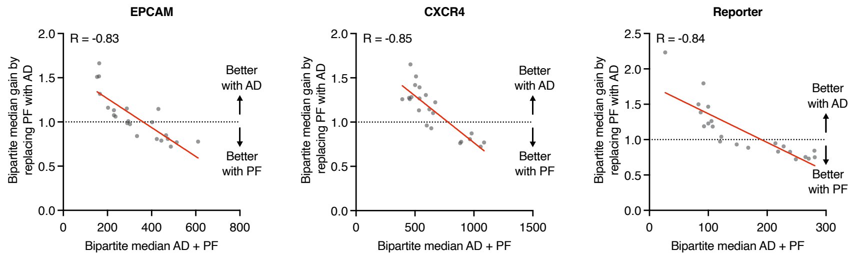

Supplementary Figure 21. Strong domains are less likely to benefit from the addition of another AD than weak domains. (a) Bipartite screen scores produced by each of 25 ADs in position 1 (y-axis) upon pairing with each of 25 ADs in position 2 (x-axis). Data are shown for *CXCR4* and the reporter with purple = lower scoring and yellow = higher scoring. ADs are ordered from weakest- to strongest-performing within the corresponding single-domain screen. Each activator is annotated based on the species it is derived from or its functionality as a human AD (white dots), viral AD (gray dots), or PF (black dots). Data are shown as the mean (n = 2 independent screen replicates). (b) Correlation between the median screen scores for activators consisting of one AD and any PF in the remaining position and the median of the change in screen score upon replacement of the PF with any AD for *EPCAM*- (left), *CXCR4*- (middle), and reporter- (right) targeting bipartite screens. Median values were calculated based on mean screen scores (n = 2 independent screen replicates). The dotted line corresponds to the point at which an AD's median performance with a PF is equal to its median performance with other ADs. Correlations were calculated using Pearson correlation coefficient (R). Source data are provided as a Source Data file (Source Data.zip).

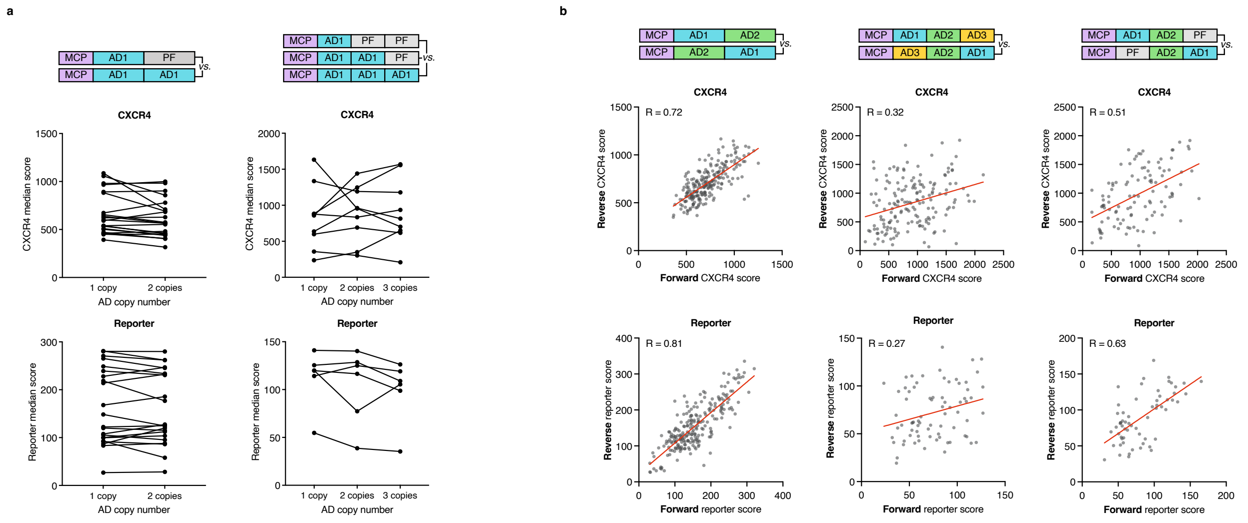

Supplementary Figure 22. Domain spatial arrangement matters more for tripartite than bipartite activators, and domain copy number usually does not affect activation. (a) Median of *CXCR4* (top) and reporter (bottom) screen scores produced by ADs present at all possible copy numbers within bipartite and tripartite screens. Values were produced by calculating the median activity of all constructs containing a given AD at the listed copy number paired with any combination of inert PF domains in the remaining positions. Median values were calculated based on mean screen scores (n = 2 independent screen replicates). (b) Correlations between screen scores for activators in forward (e.g. AD1-AD2) and reverse (e.g. AD2-AD1) orientations in bipartite and tripartite screens (left and middle), or in the tripartite screen (right) excluding any activator that does not contain one inert PF. Data, shown for *CXCR4*- (top) or reporter- (bottom) targeting screens, are presented as the mean (n = 2 independent screen replicates). Correlations were calculated using Pearson correlation coefficient (R). Source data are provided as a Source Data file (Source Data.zip).

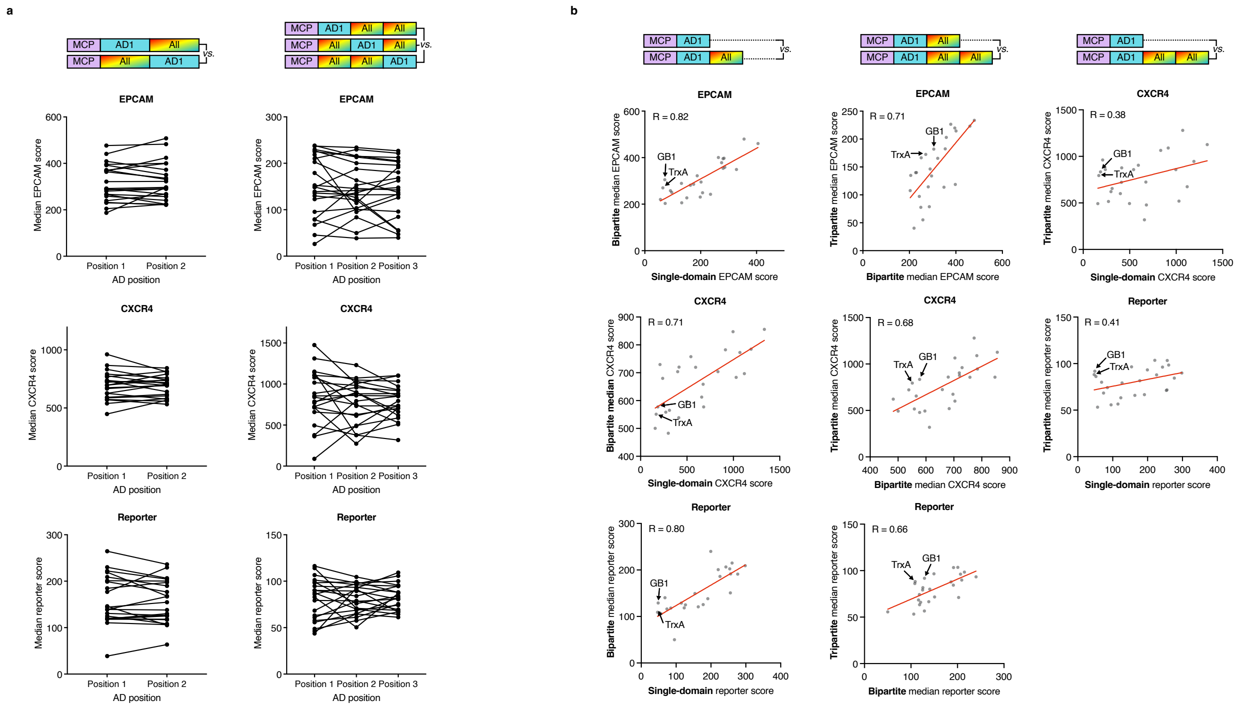

Supplementary Figure 23. Domain activation in tripartite but not bipartite systems is affected by domain positioning and adjacent elements. (a) Median of *EPCAM* (top), *CXCR4* (middle), and reporter (bottom) screen scores for each AD in all possible fusion positions. Values were produced by calculating the median of the screen scores for all constructs containing a given AD at the listed position. (b) Correlations between *EPCAM*, *CXCR4*, and reporter scores of individual ADs across all screens. Bipartite and tripartite values were produced by calculating the median scores of all constructs containing a given AD within a given screen. Arrows point to activity of PFs (GB1 and TRXA). Correlations were calculated using Pearson correlation coefficient (*R*). For all panels, median values were calculated based on mean screen scores (*n* = 2 independent screen replicates). Source data are provided as a Source Data file (Source Data.zip).

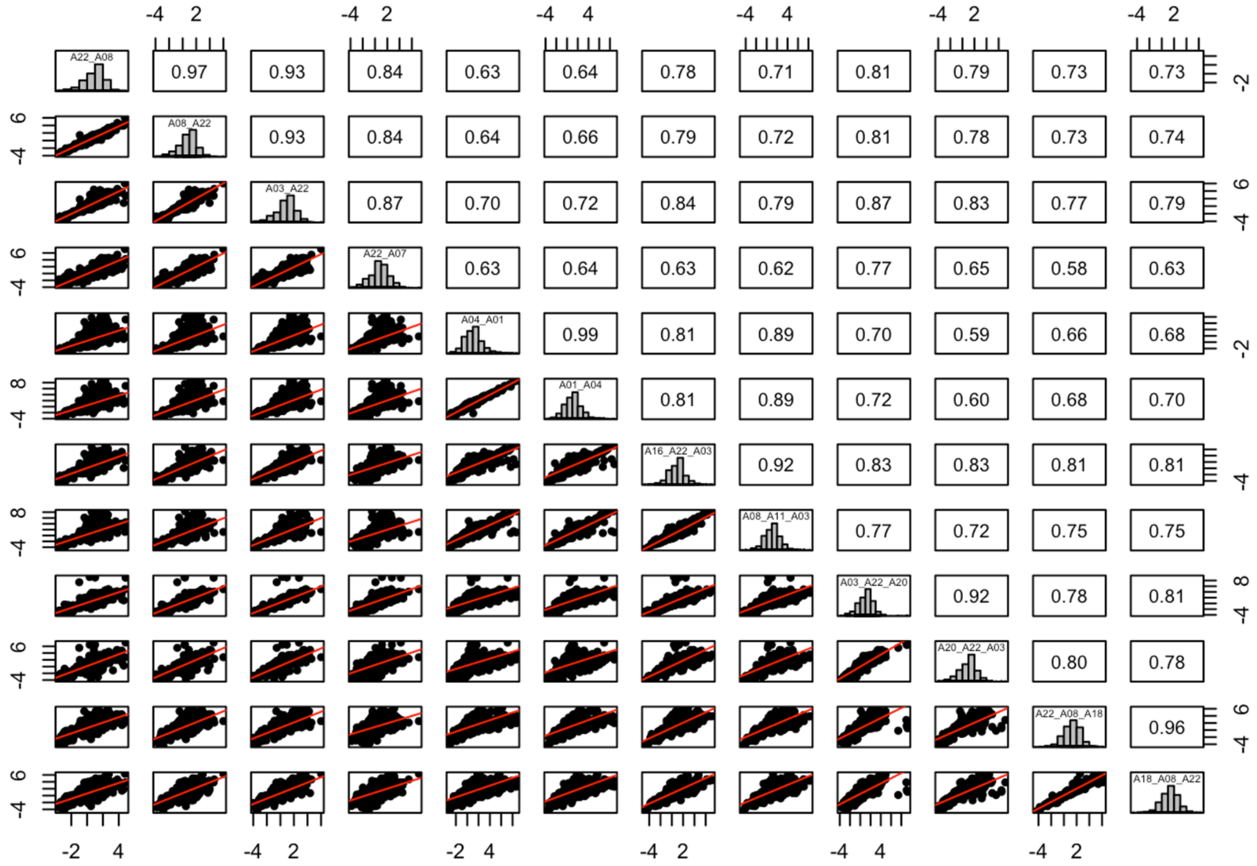

Supplementary Figure 24. Activators composed of the same domains or that display similar behavior in cells show high correlations in binding partner enrichment. Pairwise comparisons of protein binding profiles across all bipartite and tripartite activator constructs (*n* = 4 biological replicates). The grid layout shows comparisons between constructs labeled on the diagonal. Each off-diagonal plot (left of diagonal) and R value (right of diagonal) compares the construct named in its row (x-axis) against the construct named in its column (y-axis). Dots represent individual proteins' log_2(enrichment in binding over the unfused MCP control) for the two constructs being compared. Only proteins detected in all conditions are shown. Histograms along the diagonal show the distribution of log_2(enrichment in binding over the unfused MCP control) for each construct. Correlations were calculated using Pearson correlation coefficient (*R*). Source data are provided as a Source Data file (Source Data.zip).

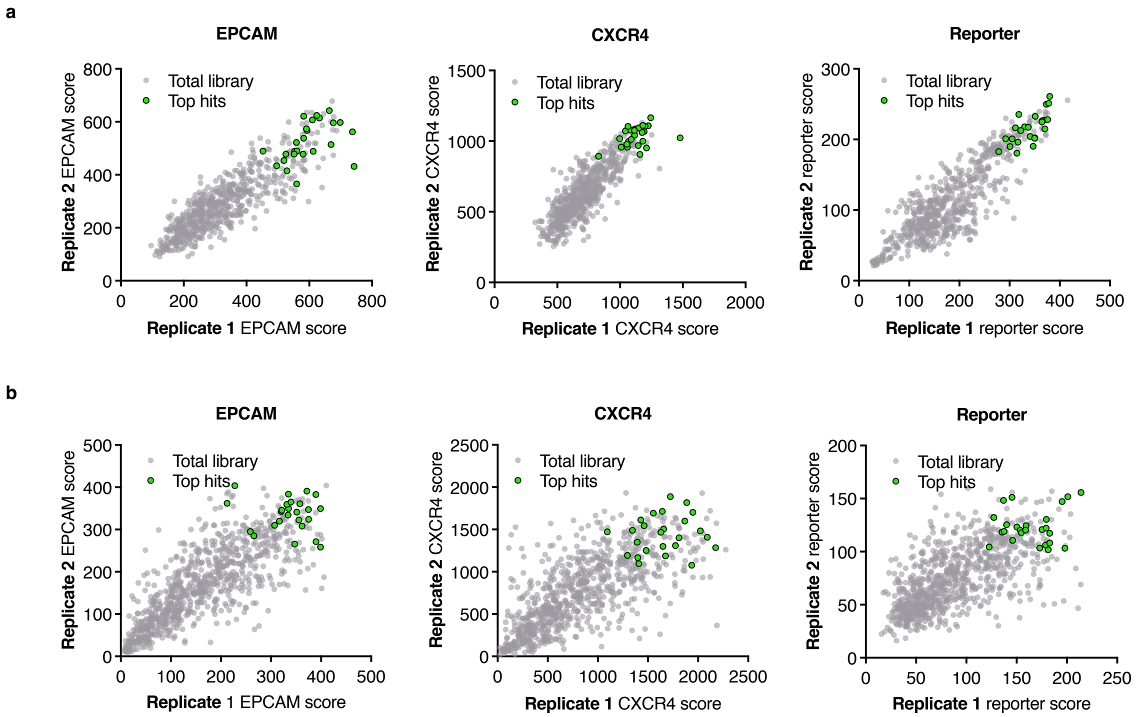

Supplementary Figure 25. Identification of top screening hits for additional workup. (a-b) Bipartite or tripartite top hits, respectively, chosen for additional workup are shown. Hits taken forward for additional testing (green) performed well relative to the total library (gray) across both replicates on three screening targets (*EPCAM*, *CXCR4*, and synthetic reporter). Source data are provided as a Source Data file (Source Data.zip).

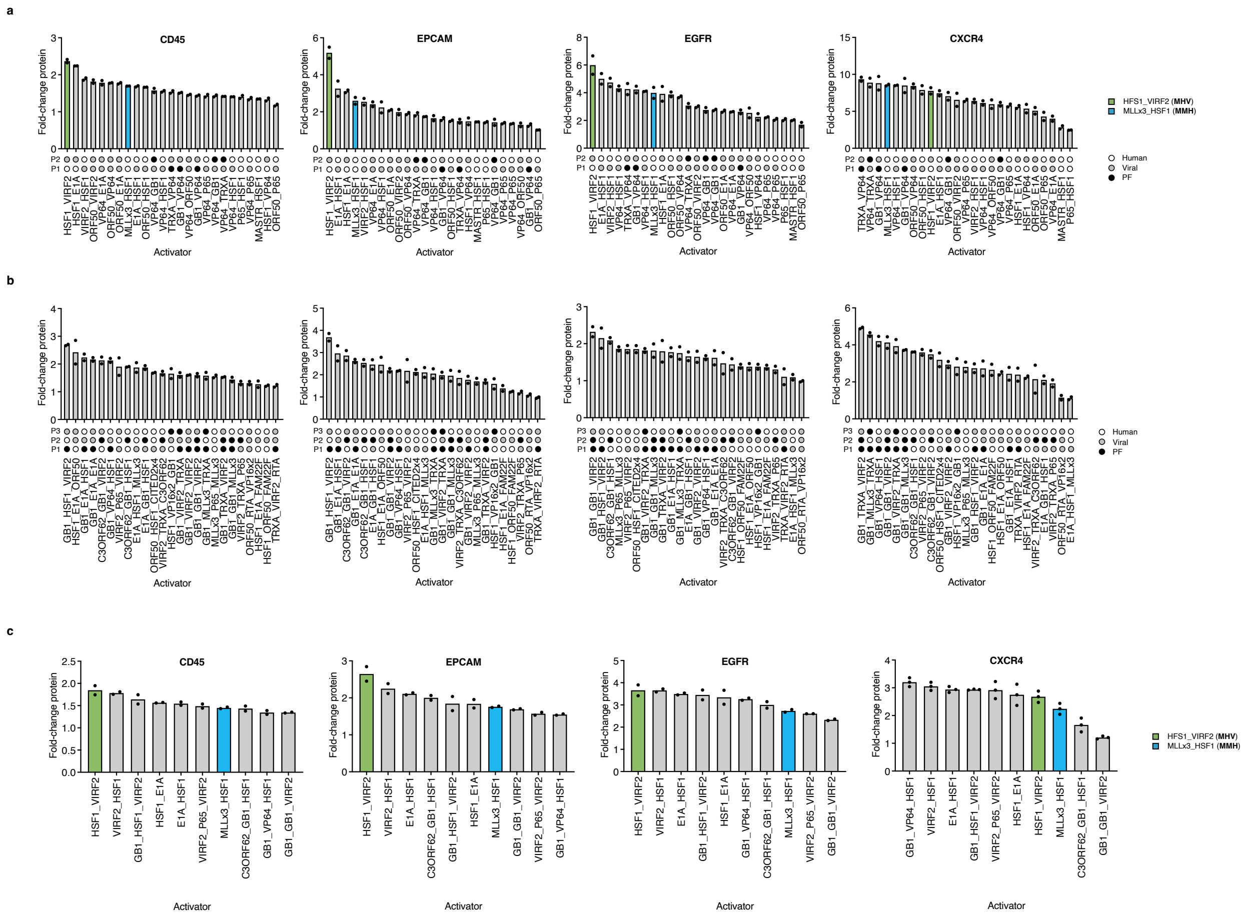

Supplementary Figure 26. MHV and MMH perform well compared to all bipartite and tripartite top hits. (a-b) Manual validation of 25 bipartite and 25 tripartite hits, respectively. Constructs were stably integrated into HEK293T cells and transfected with a mixture of gRNAs targeting four surface-protein genes, *CD45*, *EPCAM*, *EGFR*, and *CXCR4*, followed by flow cytometry to measure activation. Each activator is annotated based on the species it is derived from or its functionality as a PF (protein folder, black dots), human AD (white dots), or viral AD (gray dots). Positions within the MCP fusion (P1-P3) are shown, where P1 represents the position most proximal to MCP. (c) The same assay described in panels a and b was conducted on the top five bipartite and top five tripartite hits. For all panels, activator-produced fold-changes in target expression were normalized to a negative control line expressing MCP not fused to any protein. Black dots depict performance of each of two independent transfection replicates. For panels a and c, activation by MCP-HSF1-VIRF2 (MHV, green bar) and MCP-MLLx3-HSF1 (MMH, blue bar) are shown. Source data are provided as a Source Data file (Source Data.zip).

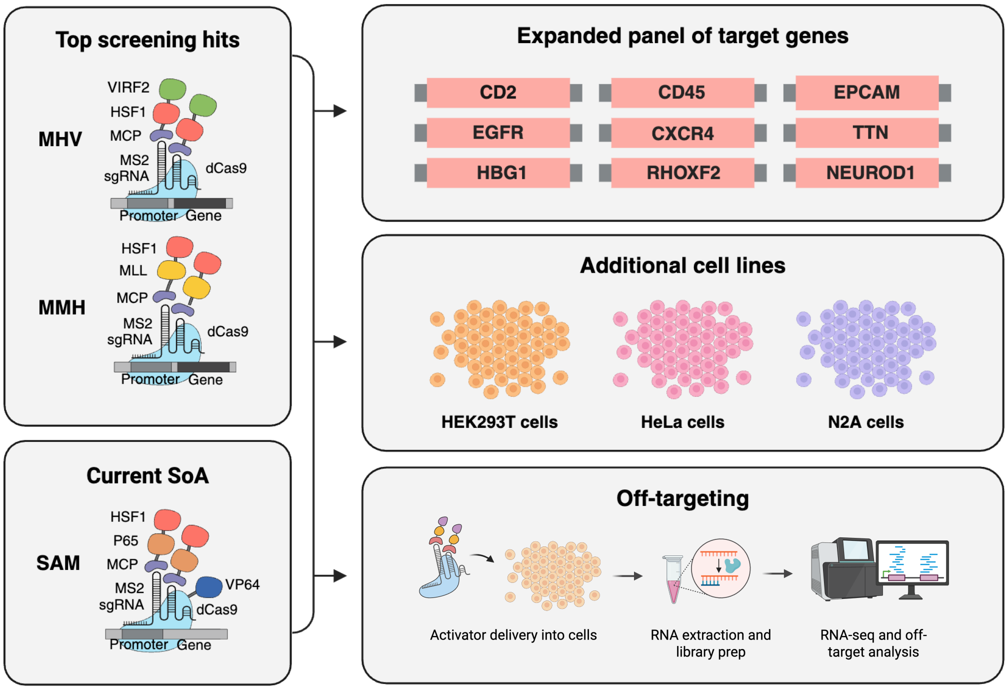

Supplementary Figure 27. Schematic depicting validation experiments (multiple targets, multiple cell lines, off-targeting) intended to evaluate the potency, generalizability, and specificity of top hits, MHV and MMH, against a current state-of-the-art MCP activator, SAM (*Created in BioRender. Giddins, M. (2025) https://BioRender.com/lgdoc71*).

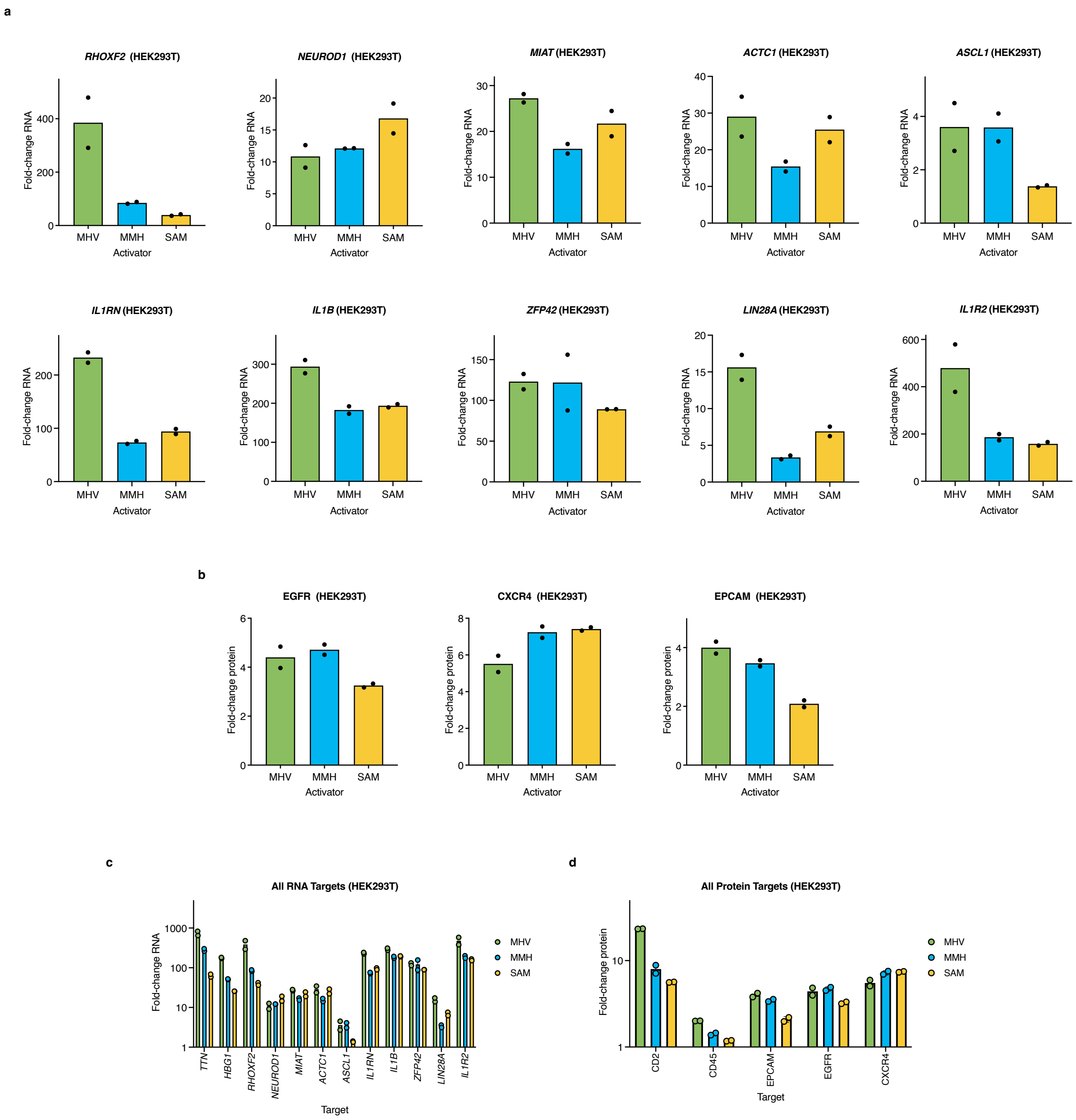

Supplementary Figure 28. MHV and MMH outperform SAM against multiple targets in HEK293T cells. (a-b) Testing activation in MHV-, MMH-, and SAM-expressing HEK293T cells against additional non-surface protein and surface protein target genes. Constructs were integrated into human HEK293T cells and transfected with a species-specific gRNA. Activation was quantified via RT-qPCR (a) or flow cytometry (b). (c-d) Summary visualization of all tested RNA (c) or protein (d) targets across MHV-, MMH-, and SAM-expressing HEK293T cells. For all panels, activator-produced fold-changes in target expression were normalized to a negative control line expressing MCP not fused to any protein. Dots depict performance of each of two independent transfection replicates. Source data are provided as a Source Data file (Source Data.zip).

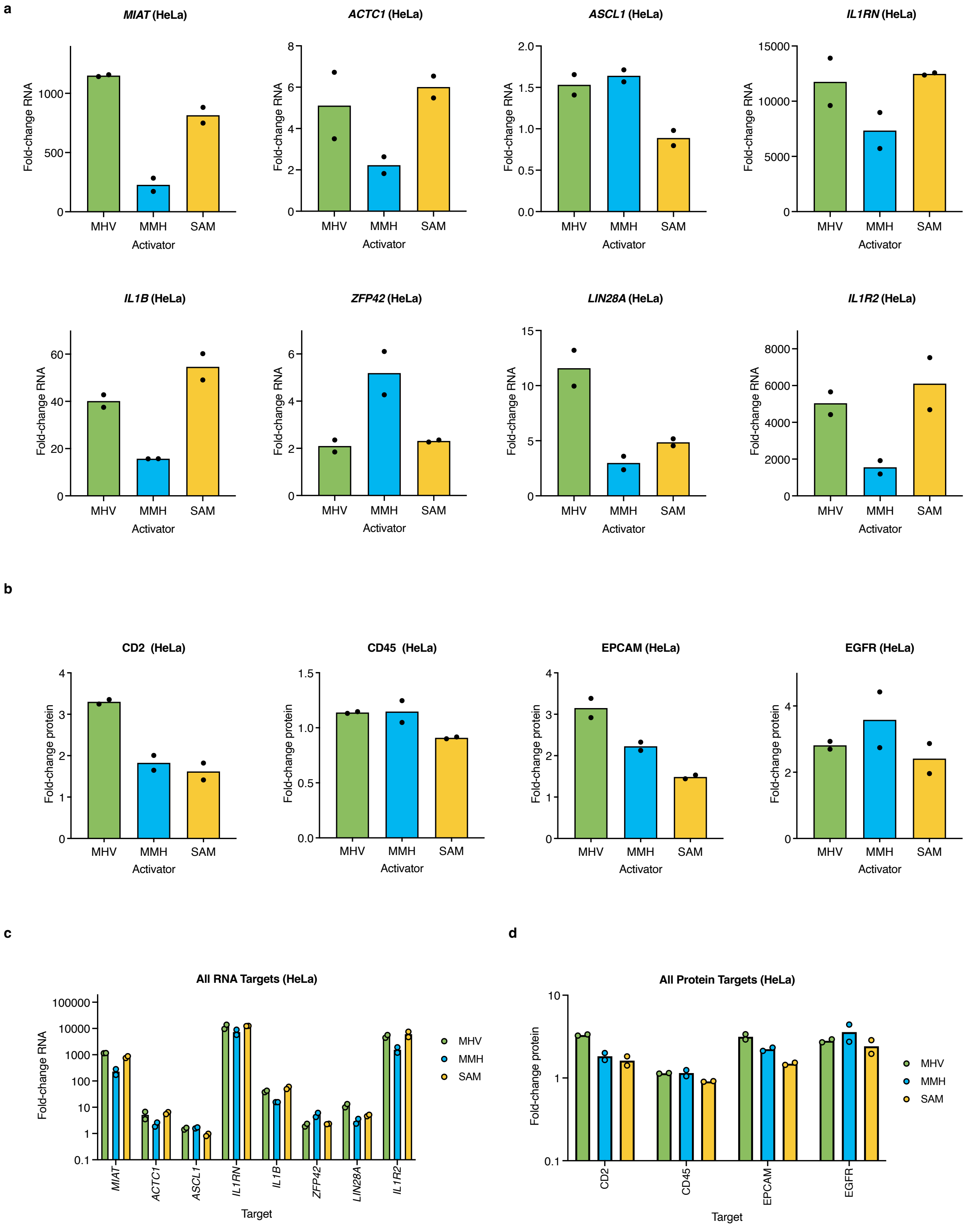

Supplementary Figure 29. MHV and MMH perform similarly to or better than SAM against multiple targets in HeLa cells. (a-b) Testing activation in MHV-, MMH-, and SAM-expressing HeLa cells against multiple non-surface protein and surface protein targets. Constructs were integrated into human HeLa cells and transfected with a species-specific gRNA. Activation was quantified

via RT-qPCR (a) or flow cytometry (b). (c-d) Summary visualization of all tested RNA (c) or protein (d) targets across MHV-, MMH-, and SAM-expressing HeLa cells. For all panels, activator-produced fold-changes in target expression were normalized to a negative control line expressing MCP not fused to any protein. Dots depict performance of each of two independent transfection replicates. Source data are provided as a Source Data file (Source Data.zip).

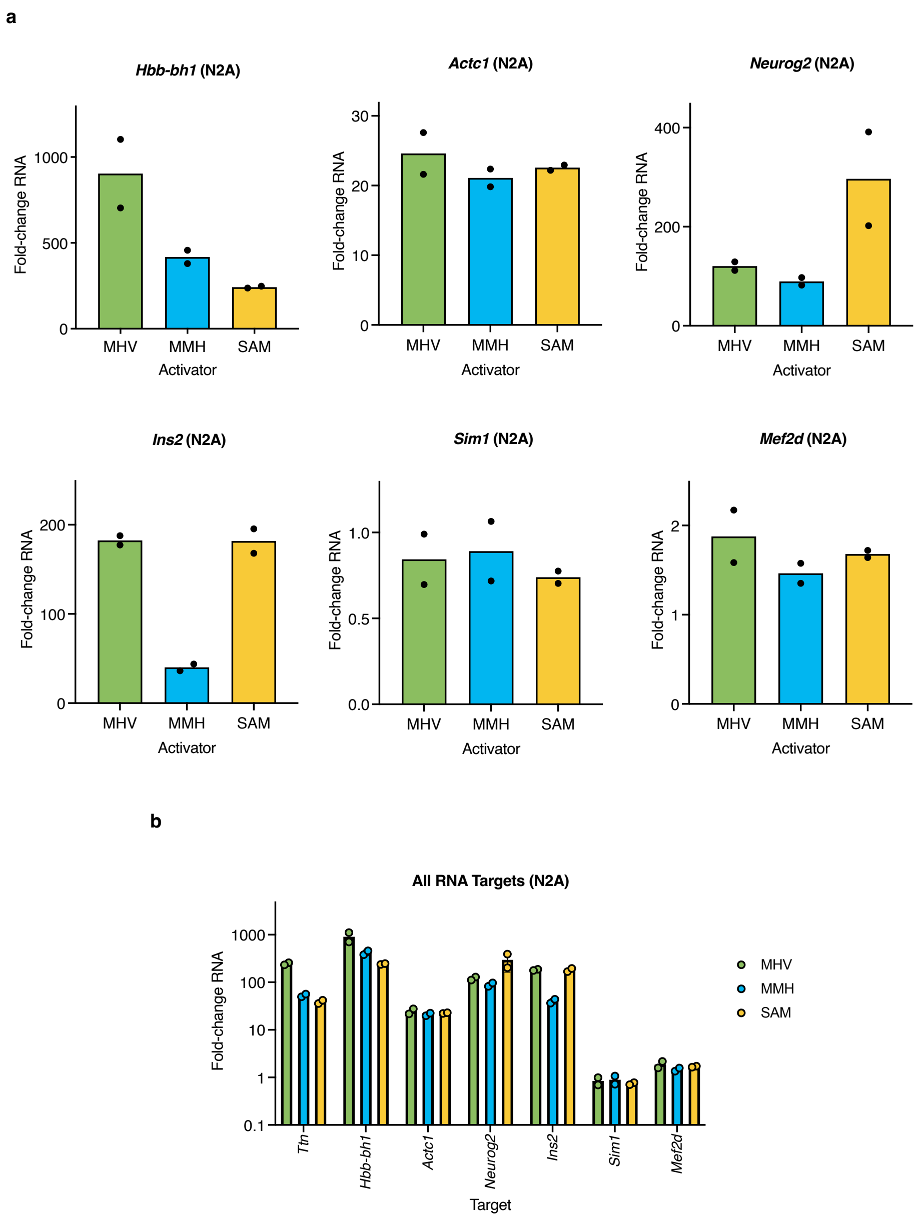

Supplementary Figure 30. MHV performs similarly to or better than SAM against multiple targets in N2A cells. (a) MHV-, MMH-, and SAM-expressing N2A cells were tested against additional non-surface protein target genes. Constructs were integrated into mouse N2A cells and transfected with a species-specific gRNA. Activation was quantified via RT-qPCR. (b) Summary visualization of all tested targets across MHV-, MMH-, and SAM-expressing N2A cells. For all panels, activator-produced fold-changes in target expression were normalized to a negative control line expressing MCP not fused to any protein. Dots depict performance of each of two independent transfection replicates. Source data are provided as a Source Data file (Source Data.zip).

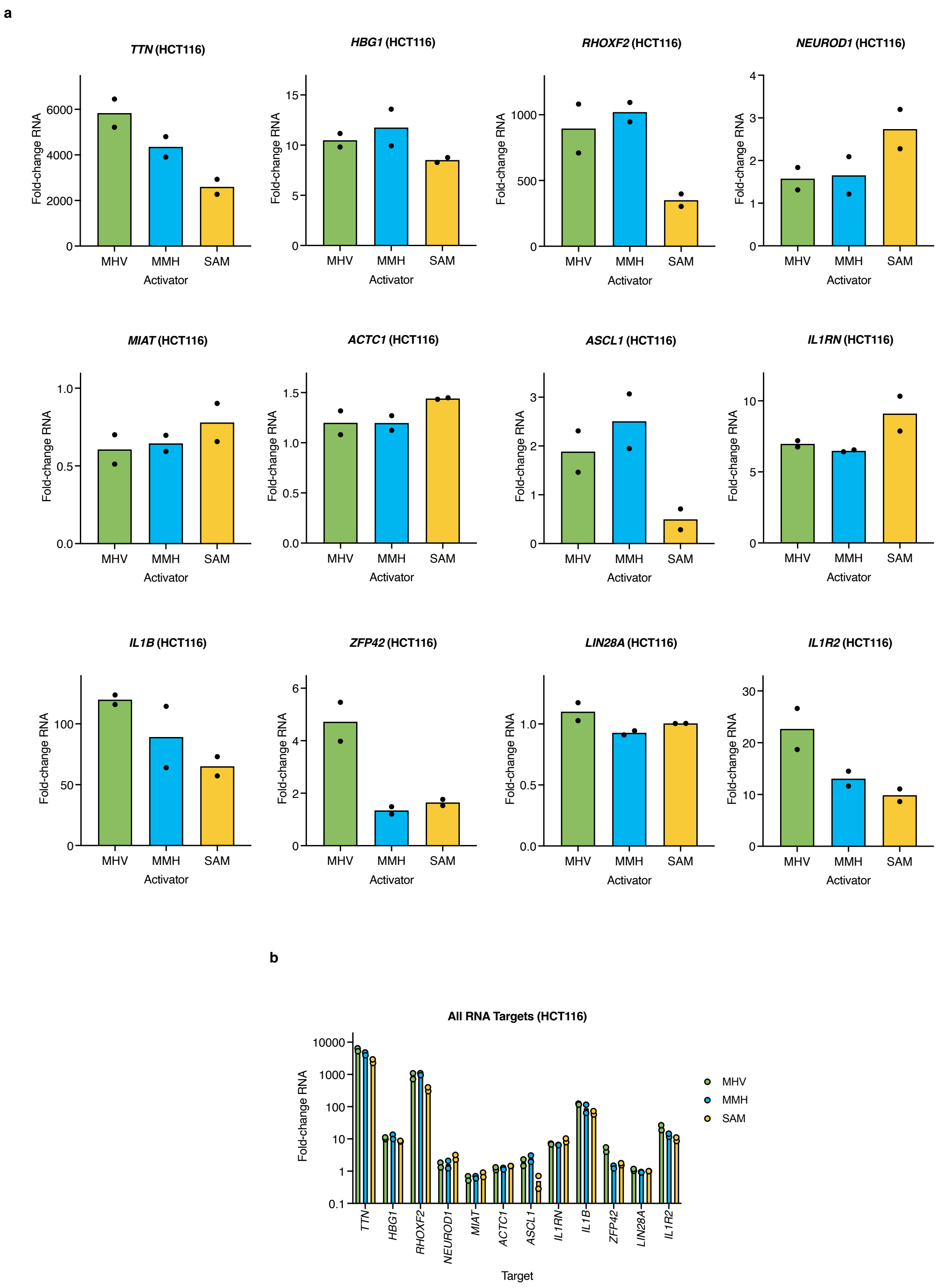

Supplementary Figure 31. (a) MHV and MMH perform similarly to or better than SAM against multiple targets in HCT116 cells. MHV-, MMH-, and SAM-expressing HCT116 cells were tested against multiple non-surface protein targets. Constructs were integrated into human HCT116 cells and transfected with a species-specific gRNA. Activation was quantified via RT-qPCR. (b) Summary visualization of all tested targets across MHV-, MMH-, and SAM-expressing HCT116 cells. For all panels, activator-produced fold-changes in target expression were normalized to a negative control line expressing MCP not fused to any protein. Dots depict performance of each

of two independent transfection replicates. Source data are provided as a Source Data file (Source Data.zip).

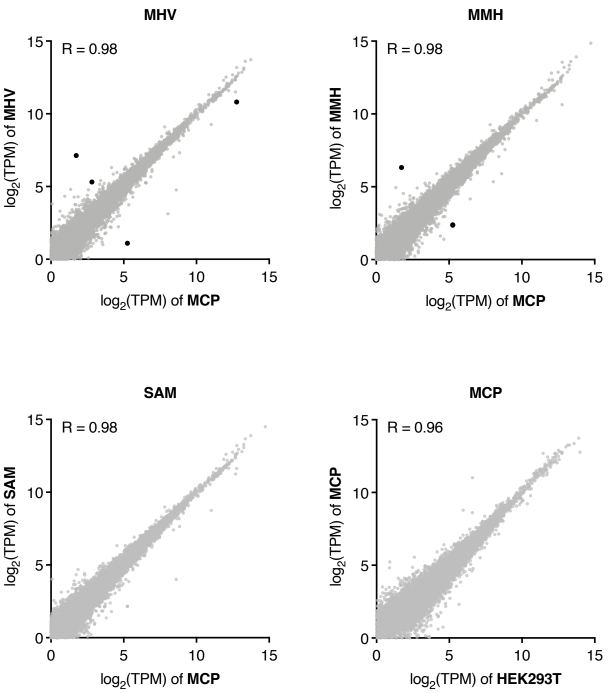

Supplementary Figure 32. Activators exhibit minimal off-target activity as compared to MCP not fused to any protein upon targeting *HBG1*. Quantifying off-target transcriptional perturbations in MHV-, MMH-, SAM-, and MCP-expressing HEK293T cells transfected with an *HBG1*-targeting gRNA. *HBG1* gene expression was below the < 1 TPM cutoff in all conditions and was therefore excluded from the plots. Transcriptional aberrations driven by MCP alone were evaluated by comparing gene expression in the MCP-expressing cell line to wildtype HEK293T cells transfected with an *HBG1* gRNA. Data are shown as the mean of $n = 2$ independent transfections. Differentially expressed genes are shown in black (defined as $|\log_2$ fold change$| > 2$ and adjusted $p < 0.05$), and all other genes are shown in gray. Correlations were calculated using Pearson correlation coefficient (R). Source data are provided as a Source Data file (Source Data.zip).

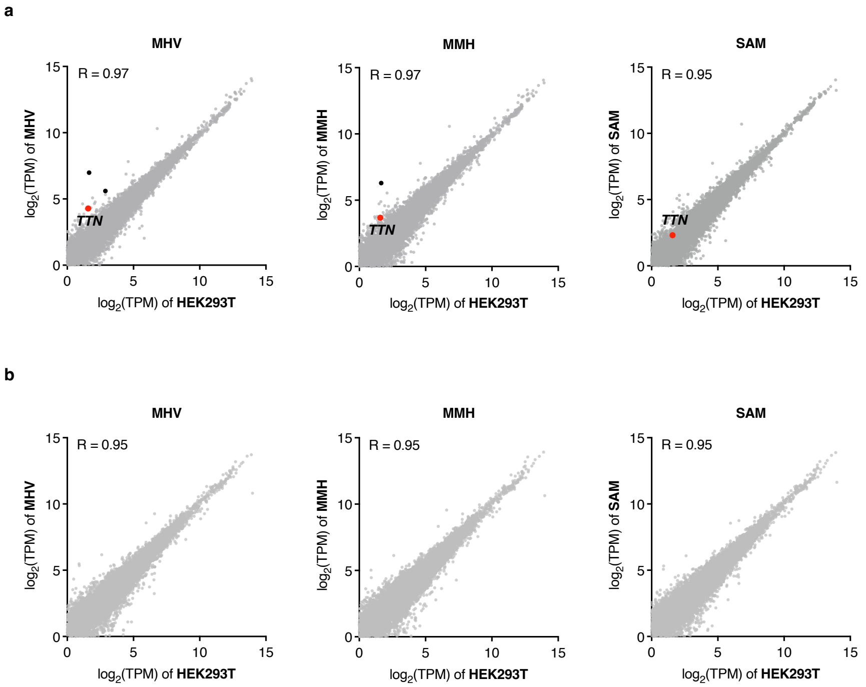

Supplementary Figure 33. Activators exhibit minimal off-target activity as compared to cells not expressing MCP. Quantifying off-target transcriptional perturbations in MHV-, MMH-, SAM-expressing HEK293T cells transfected with a (a) *TTN*- or (b) *HBG1*-targeting gRNA compared to cells absent of any MCP protein. For all panels, genes showing < 1 TPM (transcript per million) in either replicate of any construct in the given correlation were excluded before log transformation. For panel b, *HBG1* gene expression was below this cut-off and was therefore excluded from the plots. For all panels, data are shown as the mean of n = 2 independent transfections. For panel A, *TTN* (on-target gene) is shown in red. For all panels, differentially expressed genes are shown in black (defined as $|\log_2$ fold change$| > 2$ and adjusted $p < 0.05$), and all other genes are shown in gray. No genes met differential expression criteria in panel B. Correlations were calculated using Pearson correlation coefficient (R). Source data are provided as a Source Data file (Source Data.zip).

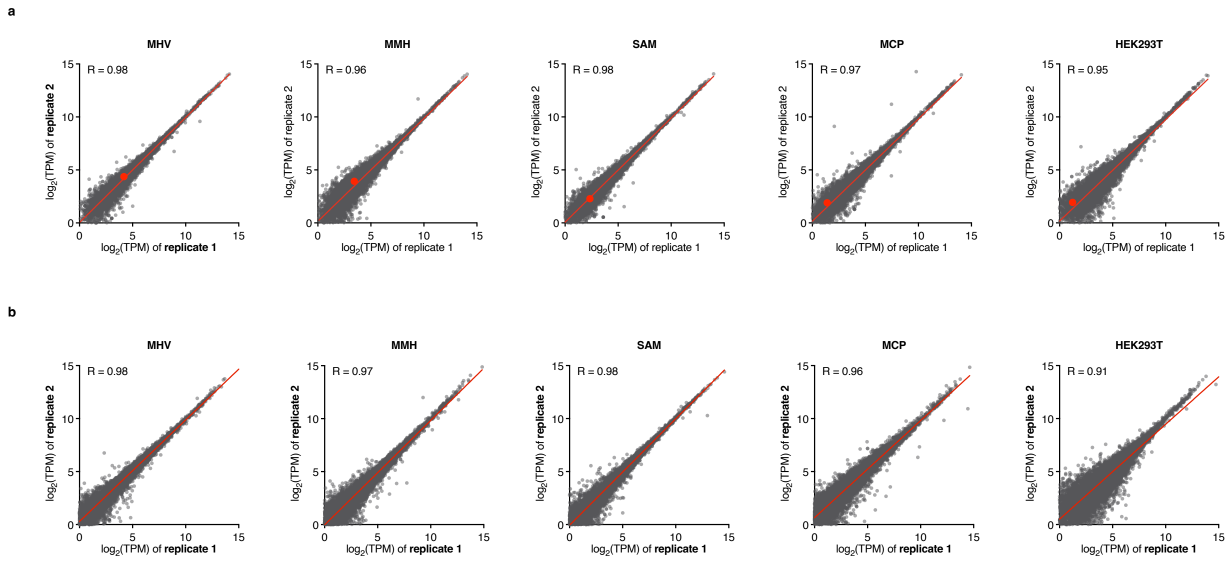

Supplementary Figure 34. Transfection replicates show high correlations in gene expression. (a-b) Correlation between *TTN* (a) or *HBG1* (b) gene expression ($\log_2$(TPM) (transcripts per million)) in transfection replicates of activator- or control-expressing lines transfected with a *TTN*- (a) or *HBG1*- (b) targeting gRNA. For all panels, genes showing < 1 TPM (transcript per million) in either replicate of any construct in the given correlation were excluded before log transformation. Correlations were calculated using Pearson correlation coefficient (*R*). For panel A, *TTN* gene expression is shown in red. *HBG1* expression is not marked in panel B because it failed to meet the TPM cutoff. Source data are provided as a Source Data file (Source Data.zip).

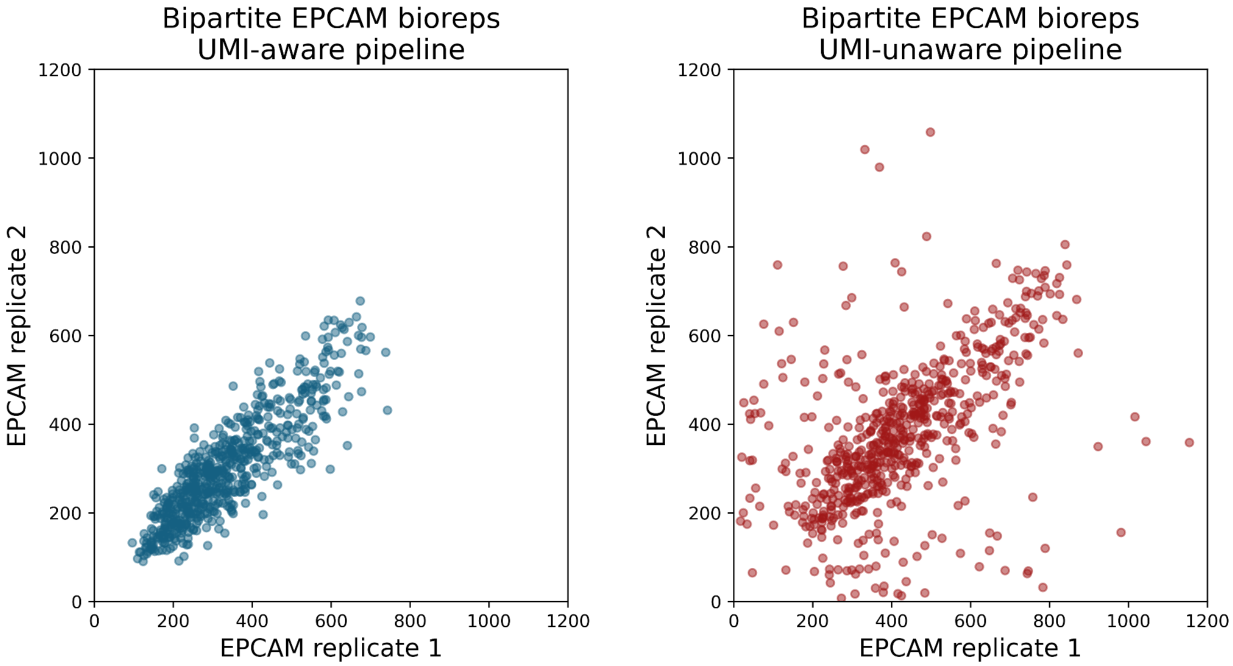

Supplementary Figure 35. UMIs enable detection and correction of noise. Comparison of biological replicates as scored from the pipeline as used (left) vs an alternative pipeline that does not incorporate UMIs (right) shows that UMI filtering removes noise that otherwise resulted in extreme outliers. UMI-aware pipeline filters out individual UMIs which are anomalously over-represented (See methods) and takes the average of the score across remaining UMIs. Axes are scaled to the same numerical values.

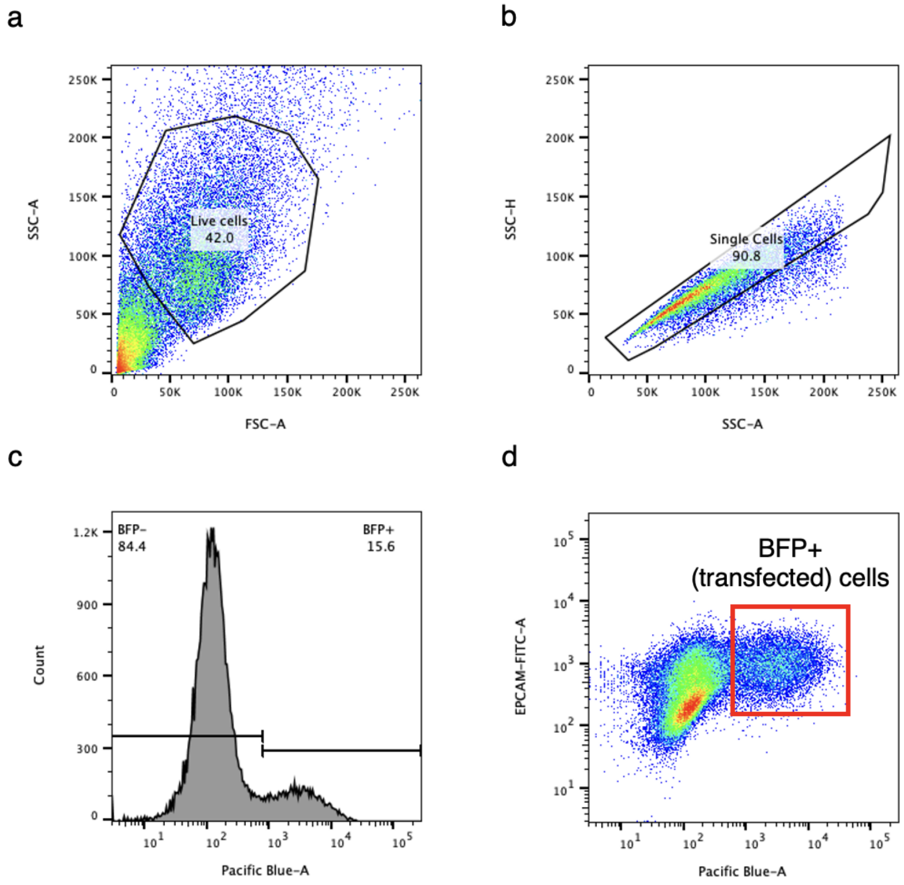

Supplementary Figure 36. Flow cytometry gating strategy for analysis of live, single, and transfected cells. (a) Live cells were gated based on side scatter area (SSC-A) and forward scatter area (FSC-A) to exclude debris. (b) Single cells were gated using side scatter height (SSC-H) versus side scatter area (SSC-A) to eliminate doublets and aggregates. (c) Transfected cells were identified by BFP expression (BFP+, right), detected in the Pacific Blue channel. BFP+ and BFP– populations were separated based on fluorescence intensity. (d) BFP+ transfected cells were analyzed for protein expression (e.g., EPCAM) using fluorescence intensity in the FITC channel. The red box indicates BFP+, EPCAM-activated cells.
