# Supplementary material for: Combinatorial protein engineering identifies potent CRISPR activators with reduced toxicity
Source: Nat Commun. 2025 Nov 20;16:11114. doi: 10.1038/s41467-025-65986-4 (PMC12706070; doi:10.1038/s41467-025-65986-4)
Supplement: Supplementary file 3 — Description of Additional Supplementary Files [file 41467_2025_65986_MOESM3_ESM.pdf]

Description of Additional Supplementary Files

File Name: Supplementary Data 1
Description: Domain origins and sequences

File Name: Supplementary Data 2
Description: Domain activity against three target genes

File Name: Supplementary Data 3
Description: PADDLE efficacy on dCas9 fusion activation data

File Name: Supplementary Data 4
Description: High-throughput screen activation scores

File Name: Supplementary Data 5
Description: High-throughput screen toxicity scores

File Name: Supplementary Data 6
Description: Activator binding profiles as determined by Msstats analysis of massspectrometry data

File Name: Supplementary Data 7
Description: Detailed MS acquisition and MaxQuant search parameters

File Name: Supplementary Data 8
Description: DNA sequences of select plasmids used in this study
